# HSG-Net: A Scheduling-Aware Heterogeneous Graph Surrogate for Aviation Sensing-Data Processing Chains

**DOI:** 10.3390/s26206662

**Published:** 2026-10-21

**Authors:** Peng Guo, Zhiqiu Huang, Pengtao Lei, Zhibin Yang

**Affiliations:** 1 College of Computer Science and Technology, Nanjing University of Aeronautics and Astronautics, No. 29 Jiangjun Avenue, Nanjing 211106, China; nwpu062475@163.com (P.G.); yangzhibin168@163.com (Z.Y.); 2 AVIC Xi’an Aeronautics Computing Technique Research Institute, No. 15, Jinye 2nd Road, Xi’an 710065, China; 3 National Institute of Excellence in Engineering, Northwestern Polytechnical University, Xi’an 710072, China; leipengtao@mail.nwpu.edu.cn

**Keywords:** aviation sensing-data processing, partition scheduling, heterogeneous graph neural networks, scheduling-aware modeling, end-to-end delay prediction, surrogate modeling

## Abstract

Efficient end-to-end timing evaluation is essential for the design and configuration of partitioned aviation sensing-data processing chains. Task activation, partition scheduling, message dependencies, and resource sharing jointly influence flow delay, while small variations in message arrival times may introduce substantial waiting across partition execution boundaries. Conventional reference simulation can capture these interactions but becomes computationally expensive when numerous candidate configurations must be evaluated. To address this challenge, this paper proposes HSG-Net, a scheduling-aware heterogeneous graph surrogate for predicting mean end-to-end delay from system configuration information. The proposed method represents tasks, partition windows, data flows, and hardware resources as interconnected entities, integrating explicit scheduling descriptors with heterogeneous relational dependencies. Graph message passing captures scheduling and resource interactions, while an adaptive direct-flow pathway preserves flow-local timing information and combines it with graph-derived representations for multi-scale delay prediction. On 7168 held-out test flows, the three-seed HSG-Net ensemble achieves a mean absolute error of 6.372 ms relative to reference-simulator labels, with 81.18% of predictions within a 10 ms absolute-error tolerance. Architectural comparisons demonstrate improved prediction over the evaluated direct-flow-only control, supporting the complementary use of relational context and flow-local information. Furthermore, HSG-Net also supports offline candidate screening through a validation-calibrated rule that allocates reference-simulation effort. The proposed approach provides an efficient, simulation-assisted method for timing evaluation and early-stage design-space exploration of partitioned aviation sensing-data processing architectures.

## 1. Introduction

Aviation sensing-data processing chains connect acquisition, preprocessing, fusion, decision, and output tasks whose timeliness depends on both execution and message delivery. In integrated modular avionics (IMA), time- and space-partitioned architectures allow multiple applications to share computing resources while maintaining isolation [1,2,3]. During architecture integration and configuration exploration, engineers need to assess alternative task activations, partition-window arrangements, processing paths, and resource mappings. These choices affect not only resource utilization but also the time required for information to propagate through a processing chain. Repeated reference simulation can evaluate candidate configurations, but its computational cost limits the number of alternatives that can be examined. This motivates a surrogate that captures scheduling-dependent delay behavior with useful predictive accuracy and lower evaluation cost, while retaining reference simulation for confirmation of selected designs.

Existing studies address partitioned-system timing and configuration development from complementary perspectives. Han et al. [4] combine partition-level schedulability analysis with communication constraints for distributed IMA systems. Fu et al. [5] use model checking to analyze ARINC 653 hierarchical preemptive scheduling. Model-based optimization methods further support the exploration of partition schedules, including multicore configurations [6,7]. Lukic et al. [8] present a model-driven approach to the automated generation and validation of ARINC 653-compliant avionics configurations. These studies connect timing analysis with avionics configuration development. HSG-Net focuses on repeated mean-delay evaluation during configuration exploration, using learned predictions to support offline candidate screening before reference-simulator confirmation. Reference simulation complements them by evaluating execution and message-transfer interactions under specified scheduling and resource configurations. Simulation acceleration through invariant-preserving transformation or parallel execution reduces the cost of individual runs [9,10], whereas surrogate modeling learns from representative evaluations to support repeated prediction queries [11,12]. In networked systems, models including RouteNet, MimicNet, DeepQueueNet, and RouteNet-Fermi demonstrate how learned surrogates can capture topology- and traffic-dependent performance [13,14,15,16,17]. These studies motivate learning-based configuration evaluation, while partitioned sensing-data processing additionally requires explicit treatment of task activation and partition service opportunities.

This scheduling dependence creates a distinctive prediction challenge. Even a small shift in message arrival can cause a downstream task to miss an eligible execution opportunity and wait for a subsequent release or partition window. Consequently, flows with similar execution demand may experience substantially different delays because of their activation types, partition phases, task dependencies, and shared resource contention. Static scheduling descriptors summarize important aspects of these mechanisms, while an explicit relational representation retains the task, flow, and resource interactions associated with each configuration. Graph neural networks provide a framework for learning from connected entities [18,19], with graph convolution and attention offering different neighborhood aggregation operators [20,21]. For this application, relational learning must distinguish message dependencies, scheduling opportunities, and hardware relationships while preserving flow-local timing information that is directly relevant to delay prediction.

We propose HSG-Net, a scheduling-aware heterogeneous graph network for mean end-to-end delay prediction in aviation sensing-data processing chains. The model integrates task timing attributes, static scheduling descriptors, and typed relations among tasks, partition windows, flows, and hardware resources. Relation-specific message passing encodes dependency, scheduling, and resource interactions, while a direct-flow pathway preserves local timing information. The resulting graph and direct-flow representations are adaptively fused for multi-scale delay decoding. This design combines explicit scheduling knowledge with relational learning to capture configuration-dependent timing behavior. The predictor further supports offline candidate screening through a validation-calibrated rule that refers selected candidates to reference simulation. The final selected designs undergo simulation-based confirmation.

The contributions are threefold. First, we develop a scheduling-aware heterogeneous representation that connects task activation, partition service opportunities, flow dependencies, and resource mappings for delay prediction. Second, we formulate a predictor that combines relational processing with direct-flow preservation and multi-scale decoding, and examine its accuracy–a complexity trade-off through architectural comparisons under a common training protocol. Third, we evaluate predictive performance across grouped scenarios and unseen hardware configurations, and demonstrate how the resulting estimates support selective simulation during offline design exploration. Independent analytical checks of selected scheduling cases and controlled label-sensitivity analyses further characterize the reference-simulation evidence and the surrogate’s response to perturbed supervision.

## 2. Problem Definition and Data Scope

### 2.1. Surrogate-Assisted Design-Space Exploration

**Figure 1:**
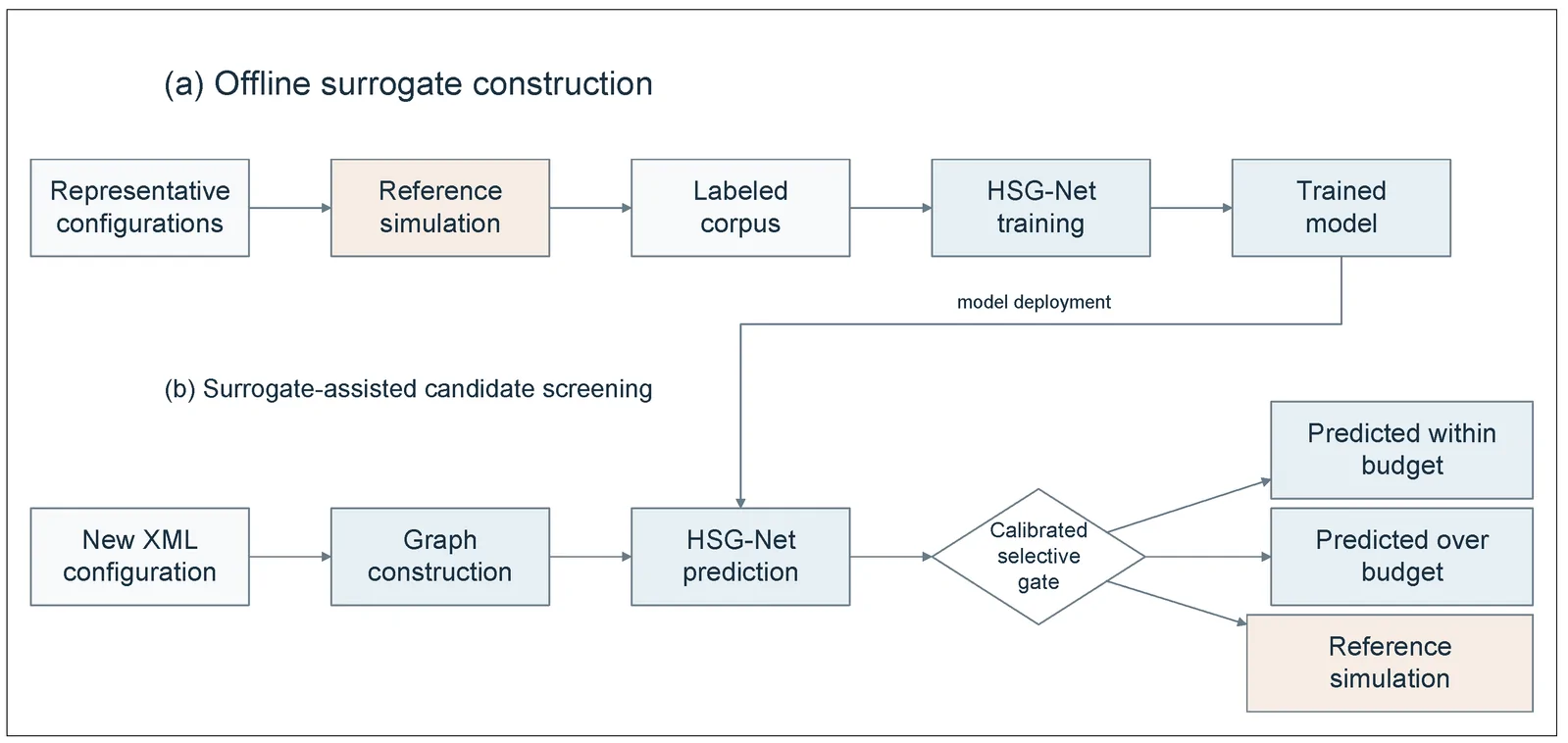
Surrogate-assisted configuration evaluation. Reference simulation supplies training labels, and HSG-Net predicts flow delays for candidate configurations during offline design exploration. A calibrated rule assigns screening decisions or refers candidates to reference simulation. Designs selected after screening undergo simulation-based confirmation.

Figure 1 summarizes the proposed workflow, consisting of offline surrogate construction and subsequent candidate evaluation. Configuration features are extracted from representative XML configurations, and corresponding flow-delay labels are obtained from reference simulation. HSG-Net learns a reusable prediction model from these paired inputs and labels. For a new candidate, configuration information is converted into a heterogeneous graph and processed by the trained model to estimate flow delays. Predictions from three models trained with different random seeds are averaged to estimate flow delay. For candidate screening, each model first takes the maximum predicted flow delay within the candidate, and the three candidate-level predictions are averaged to form the screening score. Global and domain-conditional thresholds are determined on validation configurations and held fixed during test evaluation.

Within offline design exploration, the screening rule classifies candidates as provisionally satisfying the delay budget, exceeding the budget, or requiring reference simulation. This classification allocates simulation effort across candidate configurations. Final selected designs undergo reference-simulator confirmation. Screening performance is evaluated through coverage, misclassification, and simulation cost.

Let *x* denote a candidate configuration and *S* the reference simulator. A flow represents a sequence of task executions and inter-task messages through which sensing data are processed, as follows:

(1) $$f=(\tau_{1},m_{1},\tau_{2},\ldots ,m_{L-1},\tau_{L}),$$

where $\tau_{i}$ denotes a task and $m_{i}$ a message transfer. For an observation horizon *H*, let $N_{f}\left(x;H\right)$ be the number of completed instances of flow *f*, and let $d_{f,j}$ be the end-to-end delay of its *j*th completed instance. The regression target is

(2) $$y_{f}=S_{f}\left(x;H\right)=\frac{1}{N_{f}\left(x;H\right)}\sum_{j=1}^{N_{f}\left(x;H\right)}d_{f,j},\;N_{f}\left(x;H\right)>0.$$

Thus, the target is the mean delay of completed instances within a specified observation horizon. HSG-Net predicts ${\hat{y}}_{f}=g_{\theta }\left(x,f\right)$ from configuration information. The objective is to capture scheduling-dependent delay behavior with useful predictive accuracy and lower evaluation cost.

### 2.2. Scheduling-Induced Delay Jumps

**Figure 2:**
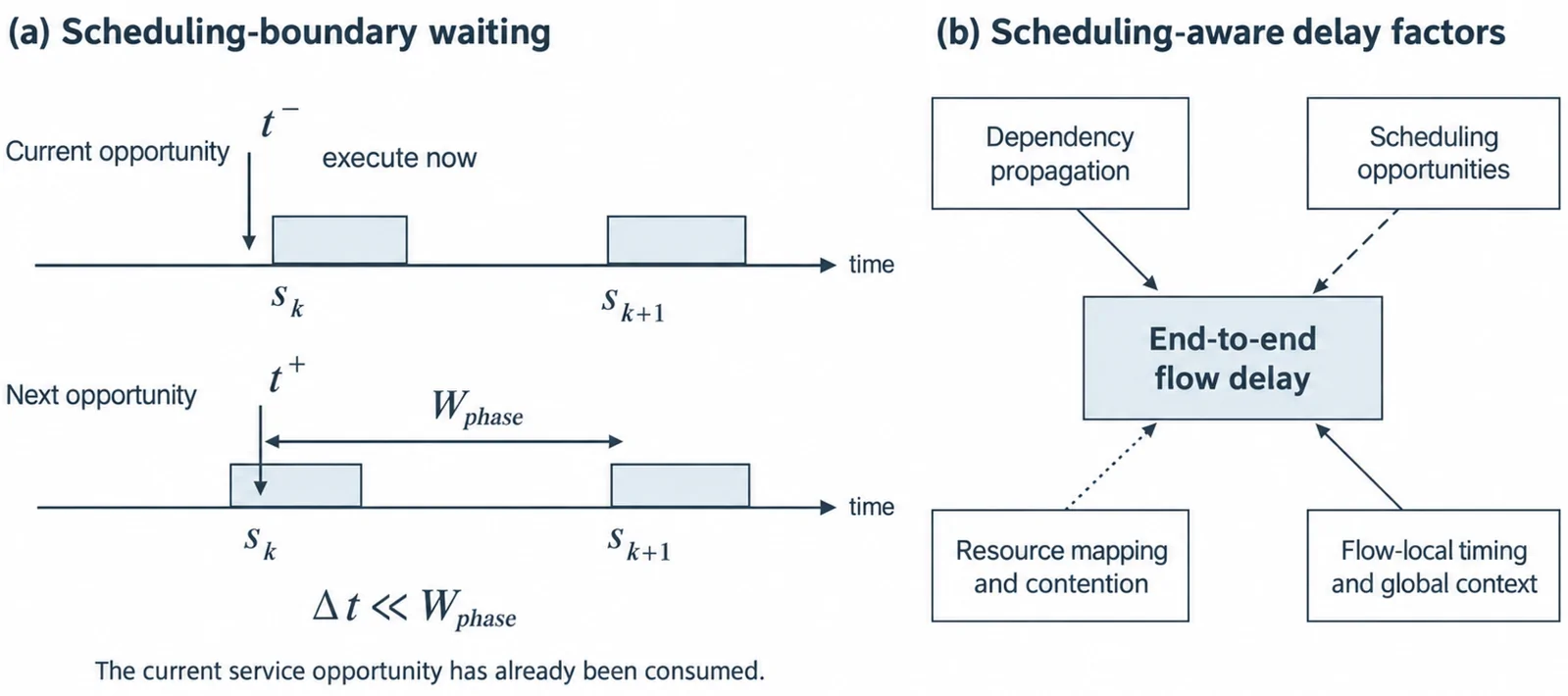
Scheduling-induced waiting and its principal influencing factors. A small arrival-time shift can defer downstream processing to a subsequent eligible service opportunity. Task dependencies, activation and partition timing, resource mapping, and contention jointly determine end-to-end flow delay.

Figure 2 illustrates why execution demand alone is insufficient to determine flow delay. Consider a downstream task with discrete eligible service opportunities at $s_{k}$ and $s_{k+1}$. An arrival at $t^{-}=s_{k}-\epsilon$ may be served at the current opportunity, whereas an arrival at $t^{+}=s_{k}+\epsilon$ may need to wait until the next eligible opportunity. The arrival-time difference is Δt=2ϵ, but the additional waiting may approach

(3) $$W_{\mathrm{phase}}\approx s_{k+1}-s_{k},\;\Delta t\ll W_{\mathrm{phase}}.$$

This example describes a boundary-sensitive scheduling situation, rather than a universal rule for every arrival within a partition window. The actual delay depends on task activation, partition availability, and competing execution demand. Periodic activation introduces release-phase and period constraints; event-triggered activation introduces dependencies on event arrival and minimum inter-arrival time. Resource mapping and contention further affect the service opportunities available along a flow. These interactions motivate a representation that combines local timing descriptors with dependency, scheduling, and resource relations.

### 2.3. Regression Target and Data Scope

The regression study considers flows with recorded mean delays no greater than 200 ms. This bound is twice the 100 ms mean-delay budget used in the primary candidate-screening case and retains substantial waiting delays caused by partition-phase alignment, period relationships, and contention. The screening budget is an engineering design constraint on mean delay, not a guarantee on every flow instance.

An additional variability filter excludes from the primary regression benchmark records satisfying both

(4) $$d_{\max}>200\;\mathrm{ms},\;\frac{d_{\max}-d_{\min}}{\bar{d}}>0.8,$$

where $\bar{d}$, $d_{\min}$, and $d_{\max}$ are the exported mean, minimum, and maximum delays. The retention criteria define the regression population using finite-horizon reference statistics.

Membership of the retained regression population is determined from reference outputs for dataset construction and evaluation, rather than from information available when screening a new candidate. The label-based filters define the flow population used in the reported evaluation. Within this population, candidate screening uses configuration-derived predictions and validation-calibrated thresholds. Table 1 shows that the mean-delay bound removes 834 records and the variability filter removes a further 88, leaving 33,264 flow records.

### 2.4. Dataset and Grouped Evaluation

The dataset contains 24,516 XML configurations and 34,186 labeled flow records before filtering. Each configuration contains one to four flows, with a separate supervised record for each flow. A base_scene_id identifies the common topology and mapping template from which related configuration variants are generated. To prevent variants of the same template from appearing in different evaluation subsets, configurations are grouped by this identifier. The retained data contain 84 training, 28 validation, and 26 test base scenes. Final test scenes are excluded from preprocessing estimation, model selection, and screening-threshold calibration.

The audited configuration files specify a 1 s observation horizon and a simulator accuracy-control setting of 100. This setting is a simulator configuration parameter, not an independently measured accuracy level. Each corpus configuration is executed once, and its flow label is computed from the completed instances observed during that execution. The exported records also contain minimum and maximum delay. Each labeled flow contains 1–48 completed instances, with a median of 19. Explicit simulator seeds are not recorded in the legacy XML corpus; neural-model seeds and dataset split identifiers are recorded for the learning experiments.

Configurations span 2–6 modules, 2–10 CPUs, 16–80 cores, 6–51 tasks, 3–16 Partition Information Table (PIT) windows, 1–16 messages, and 1–4 flows. Across flow paths, minimum task periods range from 20 to 50 ms, maximum task periods from 20 to 150 ms, and total execution demand from 4 to 20 ms.

Corpus-integrity checks found no duplicate scenario–flow keys, negative delay values, malformed XML files, or violations of $d_{\min}\leq \bar{d}\leq d_{\max}$.

### 2.5. Reference-Simulator Evidence and Evaluation Scope

The reference simulator is developed within a confidential engineering project, and its source implementation and production configurations cannot be publicly released. The supervised target is its exported finite-horizon mean delay. To provide interpretable evidence for the timing mechanisms represented by these labels, independent analytical comparisons are conducted on controlled scheduling cases. The accompanying case analyses report sanitized scenario descriptions, analytical expectations, and recorded simulator results.

The analytical checkers derive their predictions from case inputs and predefined timing recurrences without invoking the simulator, its libraries, or its internal scheduling code. Supplementary Section S9 evaluates six periodic-chain cases using two seeds, giving 12 runs and 218 completed-flow instances. All exported delays match the analytical values at the 0.001 ms output resolution, with a maximum reported absolute difference of 0.000 ms. The predeclared acceptance tolerance is 0.3 ms; this tolerance is distinct from both the output resolution and the observed discrepancy.

Supplementary Section S13 evaluates five event-triggered or mixed-activation cases. The two seeds yield 179 instance-level outputs each, giving 358 observations across 10 runs. Four cases agree with analytical point values or recurrence predictions. In the cross-window case, unfinished execution is suspended at window closure and resumed in the next partition window. Accounting for partial execution in the current window gives a deterministic completion-time range of 35–36 ms, containing recorded delays of 35.500–36.000 ms. Supplementary Section S13 provides the timing derivation and its assumptions; this range is not a statistical confidence interval. The window-capture case examines arrival before PIT opening. The case construction adopts a transfer-delay assumption of 0<τ<1 ms to examine the associated scheduling behavior.

Execution repeatability is examined separately in Supplementary Section S8 using eight configurations, six seeds, and two executions per seed. Repeated executions with the same seed produce identical outputs. These results provide evidence of execution repeatability, whereas the analytical comparisons establish agreement for the evaluated timing mechanisms. Supplementary Section S8 reports execution repeatability and historical-label comparisons, Section S11 examines observation-horizon sensitivity, and Section S12 analyzes controlled label-error propagation. Together, these analyses connect the label definition to explicit scheduling mechanisms and characterize factors affecting the interpretation of learned delay predictions.

## 3. Scheduling-Aware Heterogeneous Graph Surrogate

### 3.1. Graph Schema and Mechanism Descriptors

**Figure 3:**
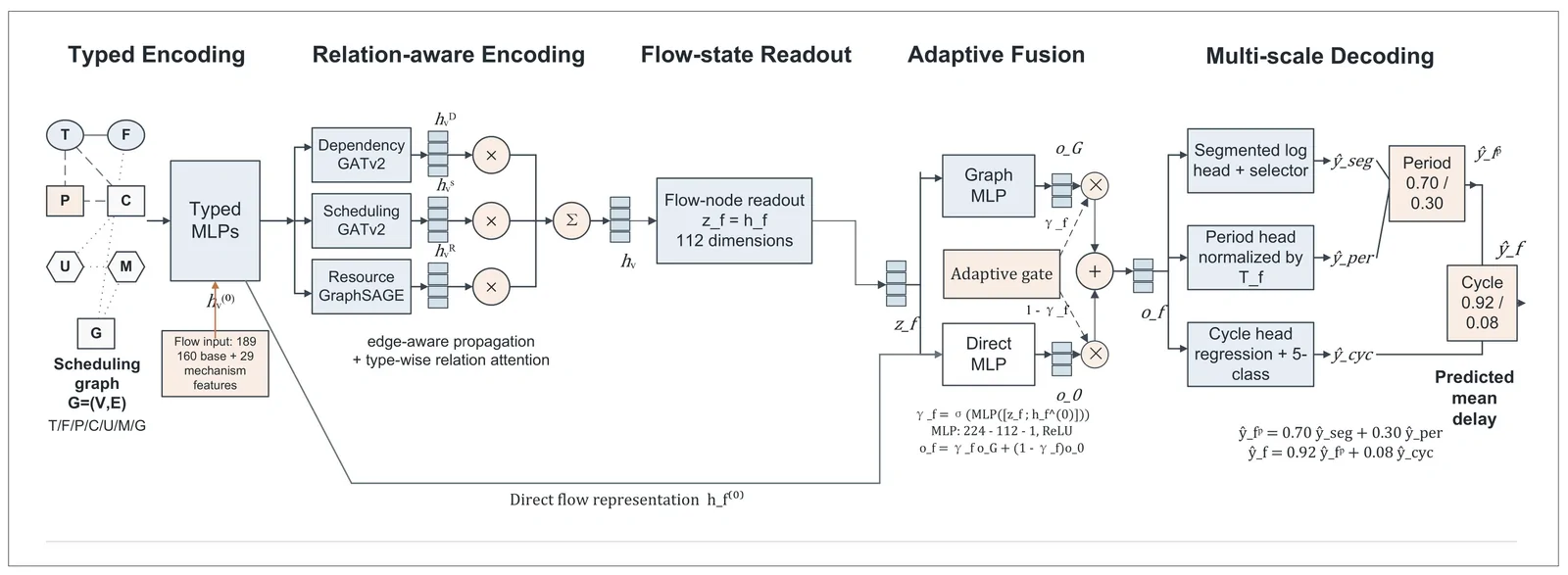
Architecture of HSG-Net. Typed encoding and relation-factorized propagation generate Flow-node representations, which are fused with direct-flow outputs before multi-scale decoding. Graph edges denote heterogeneous relations (Table S2); dashed arrows in the fusion block denote gating weights. Colors distinguish functional elements only.

Figure 3 summarizes the model architecture. HSG-Net represents configurations with variable numbers of tasks, flows, and hardware resources as a heterogeneous graph G=(V,E), without truncating the task set to a fixed size. The graph contains seven node types: Task, Flow, PIT, Core, CPU, Module, and Global. Its relations describe message dependencies, task roles within flows, task–PIT and task–core mappings, adjacent PIT windows, shared-resource contention, resource hierarchy, and global context. The graph schema comprises 35 directed relation types. The directed relation types and their reverse or companion relations are listed in Supplementary Table S2.

Scheduling information is represented through node attributes, flow-level descriptors, and typed relations. Task attributes include execution-time bounds, configured periods and deadlines, activation types, and priorities, while PIT-window attributes describe offsets, durations, and associated scheduling-load quantities. Each Flow node combines a 160-dimensional base feature vector with 29 additional scheduling-mechanism descriptors before typed encoding. Together, these inputs characterize path execution demand, period relationships, resource mapping, communication proxies, estimated arrival phase, window feasibility, priority-aware contention, and phase waiting. All input descriptors are derived from XML configuration fields; simulated delays, completed-instance counts, and execution-trace statistics are excluded from the input features. In particular, the phase-waiting descriptors are configuration-derived estimates rather than observed waiting durations.

Typed edges retain task–flow role information and encode scheduling and resource relationships. The resulting representation combines flow-local timing descriptors with the relational structure of each configuration. Table 2 summarizes the node inputs and associated relations.

### 3.2. Relation-Factorized Heterogeneous Encoder

Type-specific encoders map node features into a shared 112-dimensional hidden space, as follows:

(5) $$h_{v}^{\left(0\right)}=\phi_{\psi (v)}\left(x_{v}\right),\;h_{v}^{\left(0\right)}\in R^{112},$$

where ψ(v) denotes the node type. The encoder organizes relations into dependency, scheduling, and resource branches, denoted by *D*, *S*, and *R*. These relation families are not required to be disjoint; a relation may participate in more than one branch when it represents overlapping interactions.

The dependency and scheduling branches use edge-aware GATv2 [22], while the resource branch uses GraphSAGE [23]. Denoting the branch-specific message-passing stacks by ${\mathrm{MP}}_{k}$, the resulting states are

(6) $${\left\{h_{v}^{k}\right\}}_{v\in V}={\mathrm{MP}}_{k}\left({\left\{h_{v}^{\left(0\right)}\right\}}_{v\in V},E_{k}\right),\;k\in \left\{D,S,R\right\}.$$

Available edge attributes are supplied to the corresponding GATv2 convolutions. Each propagation layer applies a residual update, ReLU activation, and dropout. The GraphSAGE branch uses the same update structure without edge attributes. Node-type-specific attention combines the branch outputs:

(7) $$\begin{array}{cc} a_{v} & =\mathrm{softmax}\left(W_{\psi (v)}\left[h_{v}^{D};h_{v}^{S};h_{v}^{R}\right]\right), \end{array}$$

(8) $$\begin{array}{cc} h_{v} & =\sum_{k\in \{D,S,R\}}a_{v,k}h_{v}^{k}. \end{array}$$

The three attention weights are computed for each node and sum to one. This organization provides separate transformations for different relation families and adaptively combines their outputs. It draws on relation-specific transformations and semantic aggregation in heterogeneous graph learning [24,25,26].

### 3.3. Flow-State Readout and Direct-Flow Preservation

The graph representation of flow *f* is its propagated Flow-node state:

(9) $$z_{f}=h_{f}\in R^{112}.$$

The propagated Flow-node state provides the graph-derived representation for each flow, incorporating task-role information through typed task–flow relations.

A direct-flow pathway retains the initial encoding $h_{f}^{\left(0\right)}$ alongside the graph-derived representation $z_{f}$. Two structure-matched prediction heads process these representations and produce output tuples $o_{G}$ and $o_{0}$, respectively. Each tuple contains the regression values and classification logits used by the multi-scale decoder. A flow-specific fusion weight is computed as

(10) $$\gamma_{f}=\sigma \left(W_{2}\;\mathrm{ReLU}\left(W_{1}\left[z_{f};h_{f}^{\left(0\right)}\right]+b_{1}\right)+b_{2}\right),$$

where the gate maps the concatenated 224-dimensional input through a 112-dimensional hidden layer to a scalar weight. The corresponding head outputs are combined componentwise:

(11) $$o_{f}=\gamma_{f}o_{G}+\left(1-\gamma_{f}\right)o_{0}.$$

Fusion occurs before the head outputs are converted into delay estimates. The direct pathway shares the typed Flow encoder and does not use an external predictive model. This arrangement makes flow-local descriptors available alongside relational information throughout prediction. The model contains 2,687,055 trainable parameters.

The model incorporates scheduling information through flow-level descriptors and relational learning. Mechanism descriptors are encoded in the initial Flow representation, while task–flow and contention relations contribute to message passing. Partition-assignment and partition-sequence relations update Task-node representations for auxiliary task prediction. The flow-delay heads use the propagated Flow-node state and its initial encoding through the adaptive fusion defined above.

### 3.4. Multi-Scale Delay Decoding and Training Objective

The decoder represents delay at segment, period, and cycle scales. For segment-based prediction, a low/middle/high classifier selects a segment-specific log-delay regressor. The segment boundaries are the training-set median and 90th percentile, 80.278 and 115.588 ms, respectively.

A period-normalized head predicts delay relative to a representative period $T_{f}$. This period is taken from the minimum scenario major-frame value when available, followed by the minimum path period, the destination-task period, and a 25 ms fallback. A cycle-based head combines regression with classification of $y_{f}/T_{f}$ into five intervals: [0,1.25], (1.25,2.5], (2.5,4.5], (4.5,8], and (8,∞).

Let ${\hat{y}}_{f}^{seg}$, ${\hat{y}}_{f}^{per}$, and ${\hat{y}}_{f}^{cyc}$ denote the three estimates decoded from $o_{f}$ and expressed in milliseconds. Their combination is

(12) $${\hat{y}}_{f}^{p}=0.70{\hat{y}}_{f}^{seg}+0.30{\hat{y}}_{f}^{per},$$

(13) $${\hat{y}}_{f}=0.92{\hat{y}}_{f}^{p}+0.08{\hat{y}}_{f}^{cyc}.$$

The effective segment, period, and cycle weights are therefore 0.644, 0.276, and 0.080. For the three-seed ensemble, the final decoded flow-delay predictions are averaged, as follows:

(14) $${\hat{y}}_{f}^{ens}=\frac{1}{3}\sum_{j=1}^{3}{\hat{y}}_{f}^{\left(j\right)}.$$

Training combines regression, classification, and auxiliary task objectives. Let $L_{raw}$ and $L_{seg,r}$ denote the raw and selected-segment log-domain Huber losses, $L_{per}$ the period-normalized log-domain Huber loss, $L_{seg,c}$ and $L_{cyc,c}$ the classification losses, $L_{cyc,r}$ the cycle-regression loss, $L_{ms}$ a period-normalized millisecond Smooth-$L_{1}$ loss, and $L_{task}$ the auxiliary task loss. The training objective is

(15) $$L_{base}=0.45L_{raw}+0.55L_{seg,r},$$

(16) $$\begin{array}{ccc} L & = & 0.60L_{base}+0.40L_{per}+0.12L_{seg,c}\\ & & +0.10\left(L_{cyc,r}+0.4L_{cyc,c}\right)\\ & & +0.08L_{ms}+0.08L_{task}. \end{array}$$

Log-domain targets use the log(1+·) transformation and are inverted using exp(·)−1, with the corresponding period rescaling where applicable. The log-domain Huber threshold is 0.35, and the period-normalized millisecond Smooth-$L_{1}$ loss uses β=1.0. The auxiliary Task target is the recorded fraction of completed task responses exceeding the configured deadline and is fitted by mean squared error. This objective supplies task-level supervision during training; candidate screening uses the decoded flow-delay predictions. Preprocessing statistics, decoder thresholds, and model selection exclude the final test scenes.

### 3.5. Selective Candidate Screening

Candidate screening evaluates configurations against a prescribed mean-delay budget *B*. Predictions from three independently seeded models are aggregated into a candidate-level score, and validation-calibrated thresholds determine the allocation of reference-simulation effort. Ensemble and selective prediction provide the methodological context for this procedure [27,28,29].

For candidate *c*, let $F_{c}$ denote its retained flows within the regression scope. Its reference score is the largest mean flow delay:

(17) $$Y_{c}=\max_{f\in F_{c}}y_{f}.$$

Each model first produces a candidate-level prediction, after which the three predictions are averaged, as follows:

(18) $${\hat{Y}}_{c}^{\left(j\right)}=\max_{f\in F_{c}}{\hat{y}}_{f}^{\left(j\right)},\;{\bar{Y}}_{c}=\frac{1}{3}\sum_{j=1}^{3}{\hat{Y}}_{c}^{\left(j\right)}.$$

Thus, the within-candidate maximum is computed before averaging across models. The resulting ${\bar{Y}}_{c}$ is the screening score.

Let α denote the empirical validation false-feasible target. For a validation subset V, a threshold t≤B is eligible when at least one candidate has ${\bar{Y}}_{c}\leq t$ and

(19) $$\frac{\sum_{c\in V}1\left({\bar{Y}}_{c}\leq t\right)1\left(Y_{c}>B\right)}{\sum_{c\in V}1\left({\bar{Y}}_{c}\leq t\right)}\leq \alpha .$$

The largest eligible observed score is selected. Tied scores receive the same decision. If no threshold is eligible, no candidate is assigned to the within-budget category by that rule.

Two representative operating points are examined. The low-observed-error point uses a global threshold with α=0.01. The efficiency-oriented point uses α=0.10 and calibrates separate thresholds for hardware domains containing at least 30 validation candidates; other domains use the global threshold calibrated at the same α. Let $t_{c}$ denote the applicable global or domain-specific threshold. The decision is

(20) $$d\left(c\right)=\left\{\begin{array}{cc} \mathrm{predicted}\;\mathrm{within}\;\mathrm{budget}, & {\bar{Y}}_{c}\leq t_{c},\\ \mathrm{predicted}\;\mathrm{over}\;\mathrm{budget}, & {\bar{Y}}_{c}>B,\\ \mathrm{refer}\;\mathrm{to}\;\mathrm{simulation}, & \mathrm{otherwise}. \end{array}\right.$$

All thresholds are fitted on validation configurations and fixed for test evaluation. The empirical target α specifies the validation selection criterion, not a guaranteed test error rate. The two representative points form part of an exploratory comparison of global and domain-conditional rules at several validation targets.

Automatic coverage is the fraction assigned to either predicted decision category. A false-feasible decision occurs when a candidate is predicted within budget but $Y_{c}>B$, with rate

(21) $$r_{\mathrm{FF}}=\frac{N_{\text{false-feasible}}}{N_{\mathrm{predicted}\;\mathrm{within}\;\mathrm{budget}}}.$$

A false rejection occurs when a candidate is predicted over budget despite $Y_{c}\leq B$. We additionally report the fraction of true budget violations predicted within budget, $N_{\text{false-feasible}}/N_{Y_{c}>B}$, to distinguish conditional acceptance error from the proportion of violations missed. These quantities, together with referral cost, characterize the screening trade-off. Calibration and test evaluation use disjoint base-scene groups. Screening uses configuration-derived predictions; reference labels are used to calibrate thresholds and evaluate decisions. The final selected designs undergo reference-simulator confirmation.

## 4. Experiments and Results

### 4.1. Experimental Setup and Metrics

Grouped testing evaluates new base scenes while allowing hardware-domain categories to recur across splits. Strict domain holdout asks a different question: whether the model transfers to a module-count/CPU-count category absent from training and validation. Consequently, the two protocols measure distinct forms of generalization and their scores should not be interpreted as repeated measurements of an identical task.

HSG-Net uses a 112-dimensional hidden representation, two message-passing layers, four attention heads, dropout 0.12, and batch size 96. Models are optimized with AdamW using learning rate $4.5\times 10^{-4}$, weight decay $1.5\times 10^{-4}$, and gradient-norm clipping at 1.0. Training runs for at most 36 epochs with an early-stopping patience of 12 epochs; the checkpoint with the lowest validation MAE is restored. HSG-Net is trained with three random seeds: 20260903, 20260913, and 20260923. Stochastic neural baselines are also repeated three times and selected only by validation MAE. All experiments were run with Python 3.12.0, PyTorch 2.13.0, PyTorch Geometric 2.8.0, scikit-learn 1.9.0, XGBoost 3.3.0, and LightGBM 4.6.0 on an Intel Core i5-11400H CPU and an NVIDIA GeForce RTX 3050 Laptop GPU.

The main metric is mean absolute error (MAE):

(22) $$\mathrm{MAE}=\frac{1}{N}\sum_{i=1}^{N}\left|{\hat{y}}_{i}-y_{i}\right|.$$

We also report RMSE, $R^{2}$, SMAPE, and the 10 ms tolerance-hit rate, defined as follows:

(23) $$A10=\frac{1}{N}\sum_{i=1}^{N}1(|{\hat{y}}_{i}-y_{i}\left|\;\leq 10\;\mathrm{ms}\right).$$

A10 denotes a tolerance-hit rate and is not a classification metric. Confidence intervals are computed using grouped bootstrap over base scenes.

The comparison includes Ridge Regression, Histogram GBDT, Random Forest, Extra Trees, XGBoost, LightGBM, MLP, GCN, GraphSAGE, and GAT. All baseline models are provided with the same available configuration and scheduling information as HSG-Net, including task timing attributes, partition-window parameters, flow dependencies, and hardware resource mappings. Conventional tabular baselines use vectorized representations of these inputs, while generic graph baselines encode the corresponding entities and relations. All methods share the grouped split, target scope, and final test-flow keys. Hyperparameters are fixed before test evaluation: tree ensembles use 220–420 estimators, XGBoost uses depth 5 and learning rate 0.04, LightGBM uses 31 leaves and learning rate 0.035, the MLP uses hidden widths 96 and 48, and generic GNNs use two propagation layers with validation-based early stopping.

The experimental implementations use scikit-learn for conventional estimators [30], XGBoost and LightGBM for the corresponding boosting baselines [31,32], and PyTorch Geometric for graph learning [33]. The primary paired comparisons and label-error analysis use 10,000 bootstrap resamples of complete base scenes, preserving within-scene dependence. Reported MAE changes are calculated on the original test sample. Bootstrap means for architectural contrasts and label-error perturbations are reported separately in Supplementary Sections S10 and S12. The intervals characterize test-scene sampling uncertainty for the fitted ensembles.

### 4.2. Reference-Label Repeatability and Error-Sensitivity Analysis

The reference simulator provides mean end-to-end delay labels for HSG-Net training and evaluation. The characteristics of these labels are examined from three complementary perspectives: execution repeatability, observation-horizon sensitivity, and the propagation of supervision errors into surrogate predictions.

Supplementary Section S8 evaluates execution repeatability using eight configurations, six random seeds, and two repetitions per seed, resulting in 96 executions and 23 scenario–flow pairs. Repeated executions with identical seeds produce identical recorded statistics, indicating reproducible simulation behavior under fixed configuration and random conditions.

Across the six seeds, the coefficient of variation (CV) has a median of 0.754%, a 90th percentile of 4.020%, and a maximum of 9.615%. Approximate 95% confidence intervals for the mean across seeds have a median width of 0.861 ms and a 90th-percentile width of 5.926 ms. These statistics characterize execution variability and show relatively consistent mean-delay outputs for most evaluated scenario–flow pairs.

Supplementary Section S11 examines observation-horizon sensitivity at 1, 2, 5, and 10 s. For the evaluated stable periodic case, the maximum relative difference between the 1 s and 10 s mean delays is 1.289%, showing consistent mean-delay estimates over the tested observation horizons.

To examine the effect of supervision errors, Supplementary Section S12 evaluates HSG-Net under clean labels, −5% systematic label bias, +5% systematic label bias, and one realization of 5% zero-mean random noise. Each perturbed condition uses three independently seeded models trained under the same architecture and training protocol. The nominal test labels remain unchanged to enable direct comparison.

Under clean supervision, the HSG-Net ensemble achieves an MAE of 6.372 ms and a mean signed error of +0.417 ms. With −5% systematic label bias, MAE increases to 7.535 ms and the mean signed error shifts to −4.791 ms, indicating an overall tendency to underestimate reference delays. The original-sample MAE increase is 1.164 ms, with a paired 95% confidence interval of [0.490, 1.748] ms.

Conversely, +5% systematic label bias increases MAE to 7.781 ms and shifts the mean signed error to +3.496 ms, indicating an overall tendency toward overestimation. The MAE increase relative to clean supervision is 1.410 ms. The opposite directions of the signed-error shifts demonstrate that systematic bias in the training labels is reflected in the learned delay predictions.

Under the random-noise condition, the ensemble achieves an MAE of 6.710 ms, representing an increase of 0.339 ms relative to clean supervision. The paired 95% confidence interval is [−0.070, 0.689] ms, which includes zero. Compared with the systematic-bias conditions, this noise realization produces a smaller aggregate change in prediction accuracy.

The paired MAE differences are assessed using 10,000 base-scene bootstrap resamples. Taken together, the experiments show that most evaluated reference outputs have relatively low between-seed variability, while stable periodic timing estimates remain consistent across the evaluated observation horizons. They also demonstrate that systematic supervision bias can shift surrogate predictions in the corresponding direction. These findings support the interpretation of simulator-derived training labels and their influence on learned timing predictions. Table 3 summarizes the corresponding evidence.

### 4.3. Multi-Algorithm Prediction Fidelity

Table 4 compares the complete prediction methods under the common evaluation protocol. HSG-Net achieves a three-seed MAE of 6.799±0.196 ms, and averaging the three predictions reduces MAE to 6.372 ms. On the 7168 paired flows, its absolute-error reduction relative to Extra Trees is 5.156 ms; a base-scene bootstrap gives a 95% interval of [2.939, 6.886] ms. The conclusion is unchanged after assigning equal weight to each of the 26 test scenes: scene-balanced MAE is 6.124 ms for HSG-Net and 9.811 ms for Extra Trees, with a paired reduction of 3.687 ms [2.230, 5.095].

The HSG-Net ensemble also achieves an RMSE of 10.426 ms, an $R^{2}$ of 0.740, an SMAPE of 7.71%, and an A10 of 81.18%. Improvements in SMAPE and A10 show that the prediction advantage is reflected across multiple evaluation metrics rather than being determined solely by a small number of extreme residuals. The three-seed GraphSAGE ensemble reaches 11.869 ms MAE, providing an additional comparison with a generic graph-learning implementation. These results demonstrate the effectiveness of the scheduling-aware heterogeneous graph surrogate for end-to-end delay prediction under the evaluated configurations. The matched ablation study below further examines the contributions of individual HSG-Net modules.

**Figure 4:**
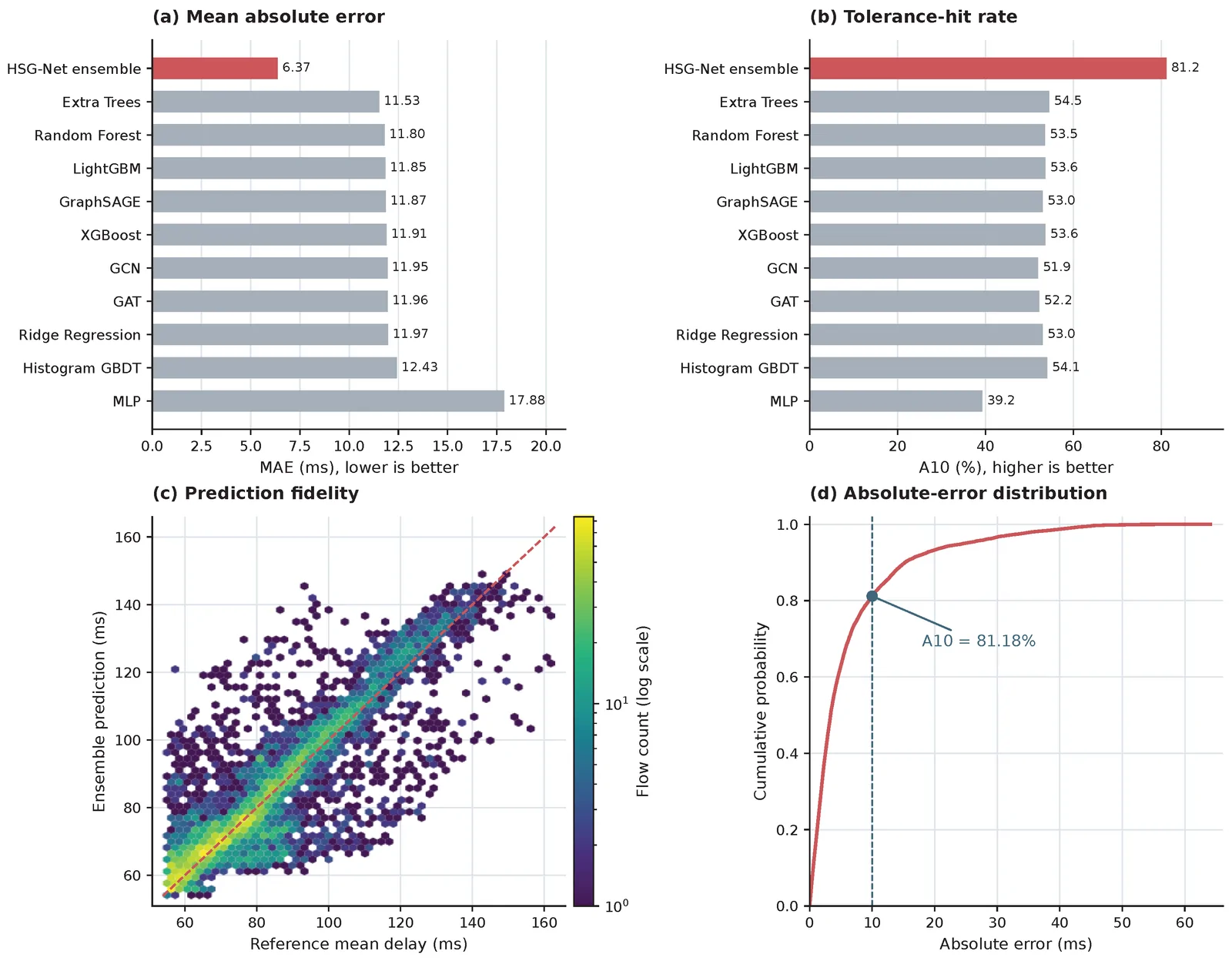
Prediction performance on 7168 common test flows. Panels (**a**,**b**) compare HSG-Net with fixed conventional baselines and three-seed neural ensembles; panels (**c**,**d**) show HSG-Net ensemble prediction density and the empirical cumulative distribution of absolute error.

Figure 4 visualizes the corresponding results.

### 4.4. Domain-Stratified and Strict-Domain Generalization

The generalization capability of HSG-Net is evaluated through domain-stratified testing and strict hardware-domain holdout. Hardware domains are defined by module count *m* and CPU count *c*. The former evaluates unseen scenes within potentially familiar hardware categories, while the latter examines transfer to hardware configurations excluded from training.

Table 5 reports HSG-Net ensemble performance across hardware domains in the grouped test set. Most domains achieve an MAE between 5 and 8 ms, indicating relatively consistent prediction accuracy across different hardware configurations. The m4_c8 and m5_c7 domains exhibit higher MAEs of 18.249 and 10.320 ms, respectively. However, each domain contains only one base scene, with 37 and 44 test flows. Their results therefore reflect individual scene characteristics rather than general performance across the corresponding domains. Negative within-domain $R^{2}$ values are interpreted alongside MAE and A10 because limited variation in reference delays can affect variance-normalized metrics.

To further evaluate transfer to unseen hardware layouts, a strict leave-one-domain-out protocol is adopted. For each target domain, its module-count/CPU-count combination is excluded from training, preprocessing estimation, validation-based model selection, and threshold calibration. Four representative domains are considered: m2_c3, m3_c5, m4_c8, and m5_c7.

As shown in Table 6, HSG-Net achieves ensemble MAEs of 7.485, 7.842, 6.474, and 8.389 ms across the four target domains, with corresponding A10 values of 78.84%, 76.57%, 85.08%, and 77.61%. HSG-Net achieves a lower MAE than XGBoost and GraphSAGE in all four evaluated domains.

These results indicate that HSG-Net can predict end-to-end delays for hardware-layout categories not encountered during training. The heterogeneous graph representation preserves task dependencies, partition scheduling relations, and hardware resource interactions across configurations of different sizes, supporting the transfer of learned timing relationships.

The m4_c8 strict-holdout target contains 583 flows but only two independent base scenes, resulting in a relatively wide scene-bootstrap confidence interval. This highlights the importance of independent scene support when evaluating cross-domain performance. Domain-stratified testing and strict-domain holdout therefore provide complementary evaluations of generalization.

**Figure 5:**
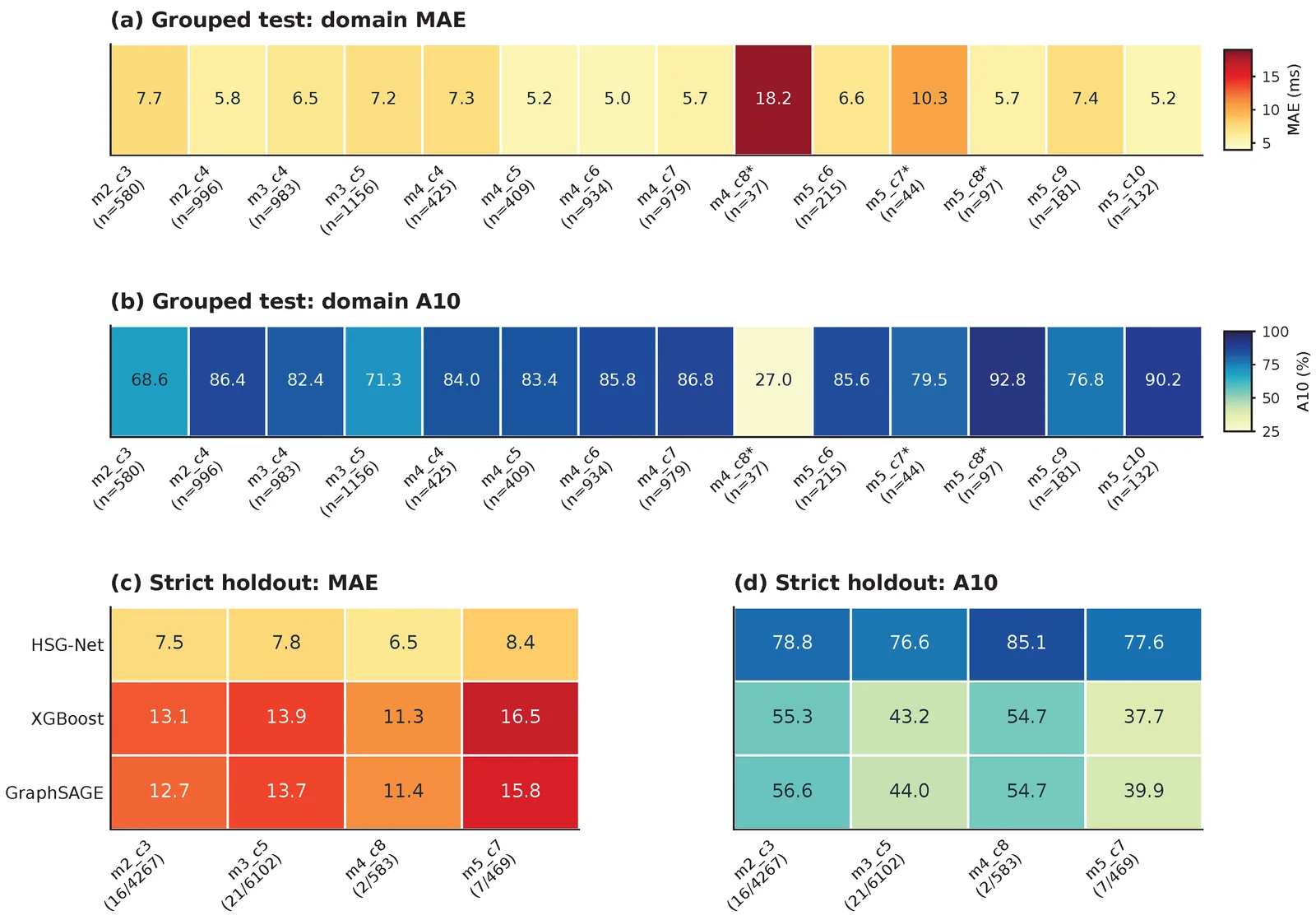
HSG-Net domain generalization. Panels (**a**,**b**) show domain-stratified ensemble MAE and A10 on the grouped test set. An asterisk (*) indicates a domain containing fewer than 100 test flows in the grouped-test split. Panels (**c**,**d**) compare ensemble MAE and A10 under strict hardware-domain holdout. Scene-bootstrap confidence intervals for the strict-domain results are reported in Table 6.

Figure 5 summarizes the domain-stratified results and strict-domain comparisons.

### 4.5. Architectural Comparisons and Module Ablation

We investigate architectural choices from two perspectives: the predictive value of graph processing and the trade-off between additional components and model complexity. Table 7 compares alternative architectures, and Table 8 reports matched deletions from the adopted HSG-Net. All configurations use the same data split, three random seeds, a maximum of 36 epochs, an early-stopping patience of 12 epochs, and checkpoint selection by validation MAE. The reported test metrics use three-seed ensemble predictions. Paired MAE differences are computed from the original predictions, with 95% intervals obtained from 10,000 bootstrap resamples of the 26 test scenes.

#### 4.5.1. Architecture Comparison

Combining graph processing with flow-level descriptors produces the clearest architecture-level improvement. The direct-flow-only alternative receives the same Flow-node descriptors but reaches an MAE of 9.953 ms, compared with 6.372 ms for HSG-Net. The paired difference is 3.581 ms [2.404, 4.740]. This result supports representing the processing chain through both flow descriptors and relationships among its constituent entities. The comparison encompasses the complete architectures: the direct-flow-only alternative also removes graph–direct fusion and task-level auxiliary supervision.

The readout comparison identifies an opportunity for simplification. Explicit source, intermediate, sink, and full-path pooling yields an ensemble MAE close to that obtained by directly reading the propagated Flow-node state. Removing this additional pooling stage reduces the parameter count by 14.60% and single-candidate inference time from 57.20 to 54.92 ms. Role pooling achieves a slightly lower mean best validation MAE (8.375 versus 8.445 ms), whereas direct Flow-node readout achieves a slightly lower ensemble test MAE (6.372 versus 6.417 ms). The paired test-MAE difference is 0.046 ms [−0.242, 0.282]. Together, these results favor direct readout as a compact implementation with similar observed prediction performance. Task roles remain represented by the typed task–flow relations used during message passing.

Relation factorization presents a different balance. Replacing the relation-family branches with a unified heterogeneous GATv2 stack reduces parameters from 2.687 to 2.214 million, but increases the mean best validation MAE from 8.445 to 9.175 ms. Measured inference times remain nearly identical, at 54.92 and 54.86 ms, respectively. HSG-Net therefore retains relation factorization for its observed validation performance at a comparable inference cost. The unified alternative preserves edge-type-specific parameters, so this comparison concerns the organization of heterogeneous propagation.

#### 4.5.2. Matched Module Ablation

The remaining deletions examine how the adopted architecture uses scheduling associations and the direct-flow pathway. Removing scheduling relations or direct-flow preservation increases the ensemble MAE by 0.080 and 0.228 ms, respectively, while removing both increases MAE by 0.253 ms. Their paired intervals span zero, and the corresponding A10 changes are small. These comparisons retain other routes for the same information: scheduling attributes remain available in node descriptors and retained edge attributes, and Flow-node features continue to participate in graph propagation after removal of the direct branch.

The complexity trade-offs also differ from the readout comparison. Scheduling-edge deletion leaves the parameterized architecture unchanged, while removing direct-flow preservation reduces parameters by approximately 4.79% and removes the direct route from the initial Flow representation to the prediction heads. Neither deletion yields a lower ensemble MAE in this comparison. The adopted configuration retains these pathways as an engineering trade-off, while implementing the larger parameter reduction provided by direct Flow-node readout.

**Figure 6:**
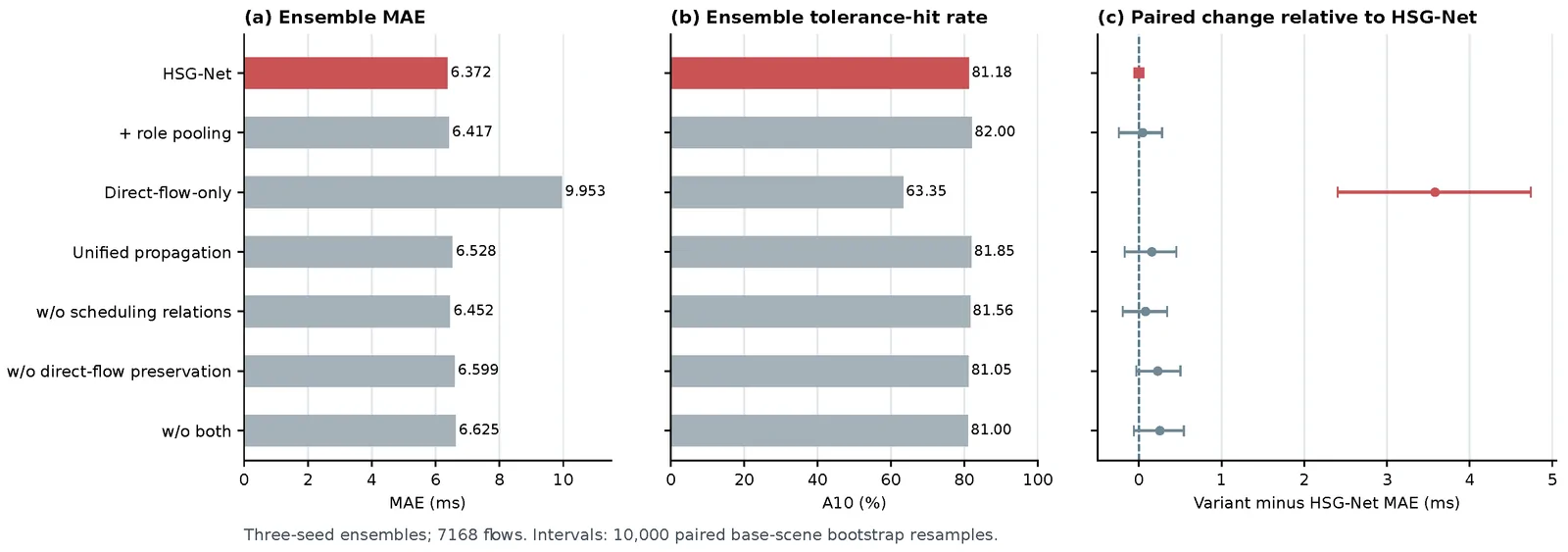
Architecture comparisons and matched module deletions. Panels (**a**,**b**) report ensemble MAE and A10; panel (**c**) shows paired MAE differences relative to HSG-Net and 95% base-scene bootstrap intervals. Positive differences indicate higher MAE. The role-pooling and direct-flow-only variants are architecture controls; the remaining variants modify the role-pooling-free architecture.

Overall, the selected configuration combines compact Flow-node readout with relation-factorized propagation, explicit scheduling associations, and direct access to flow-level information. Figure 6 summarizes the ensemble results and paired component effects. The architecture comparisons are supplementary analyses of the shared benchmark; bootstrap intervals characterize test-scene sampling uncertainty for the fitted ensembles.

### 4.6. Ensemble Dependence and Offline Candidate Screening

The three independently seeded HSG-Net models exhibit pairwise prediction correlations of 0.933–0.953 and residual correlations of 0.776–0.832. The Spearman correlation between prediction disagreement and absolute error is 0.299. These results characterize the dependence among ensemble members and indicate that their prediction errors remain correlated despite different training seeds. The ensemble uses averaged predictions for flow-delay estimation and candidate screening.

Screening thresholds are calibrated using 4732 candidates from 28 validation scenes and evaluated on 5254 candidates from 26 disjoint test scenes. Under the 100 ms mean-delay budget, 4011 test candidates satisfy the reference constraint, while 1243 exceed it. For each model, the candidate-level prediction is defined as the maximum predicted delay among its flows. The arithmetic mean of the three model-specific candidate predictions is then used as the screening score. Candidates with scores below the calibrated feasibility threshold are classified as within budget, those with scores above 100 ms are classified as over budget, and the remaining candidates are referred to reference simulation.

Two representative operating points are evaluated to examine the trade-off between screening coverage and prediction errors. The low-observed-error operating point uses a global feasibility threshold of 57.746 ms, calibrated with a 1% empirical false-feasible target on the validation set. On the test set, 181 candidates are automatically classified as within budget, with no observed false-feasible decisions. Together with 1327 automatically identified over-budget candidates, this operating point achieves 28.70% automatic coverage.

The efficiency-oriented operating point employs domain-conditional feasibility thresholds calibrated with a 10% empirical false-feasible target. Domains containing fewer than 30 validation candidates use the corresponding global threshold. This strategy automatically identifies 2835 candidates as within budget, including 81 false-feasible decisions, resulting in a 2.86% false-feasible fraction. Together with 1327 automatically identified over-budget candidates, the automatic coverage increases to 79.22%.

Further analysis of the 81 false-feasible decisions shows that their reference mean delays exceed the 100 ms budget by a median of 10.35 ms, a 90th-percentile value of 24.87 ms, and a maximum of 31.32 ms. These cases account for 6.52% of the 1243 truly over-budget candidates. Both operating points also produce 264 false over-budget decisions, corresponding to candidates that satisfy the reference budget but are predicted to exceed it.

These results quantify the trade-off between screening efficiency and decision errors in offline configuration exploration. False-feasible decisions may retain configurations that exceed the reference delay budget, while false over-budget decisions may exclude potentially useful designs. HSG-Net therefore serves as a preliminary evaluation and simulation resource-allocation mechanism. Final selected designs remain subject to reference-simulator confirmation before further engineering evaluation.

**Figure 7:**
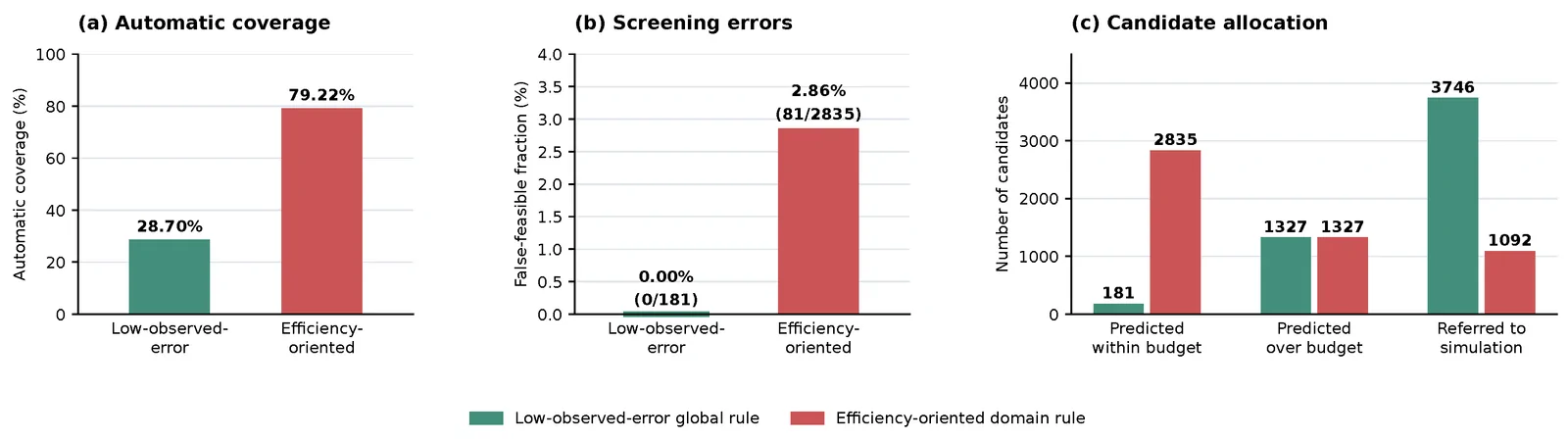
Comparison of two offline candidate-screening strategies under a 100 ms mean-delay budget on 5254 test candidates. (**a**) Automatic coverage, including candidates predicted within or over budget. (**b**) Observed false-feasible fractions among candidates predicted within budget; parentheses show the corresponding error counts and denominators. (**c**) Numbers of candidates predicted within budget, predicted over budget, and referred to reference simulation.

The two representative operating points illustrate different coverage–error trade-offs. Supplementary Section S7 reports four operating points using global and domain-conditional calibration at α=0.01 and α=0.10. Table 9 summarizes the screening outcomes, and Figure 7 illustrates the coverage–error trade-off.

### 4.7. Computational Efficiency and Break-Even Analysis

The computational efficiency of HSG-Net is evaluated through model inference time, configuration-processing overhead, reference-simulation cost, and simulation-referral frequency. For single-candidate evaluation, the measured warm inference times are 54.92 ms for a singular model and 152.26 ms for the three-model ensemble, including tensor transfer. Combining these measurements with the existing median XML parsing time of 3.56 ms and graph-construction time of 29.66 ms yields estimated pipeline times of 88.14 ms and 185.49 ms, respectively.

The median reference-simulation time is 121.00 s per configuration. Accordingly, the estimated isolated single-model evaluation speedup reaches 1372.77×. This result demonstrates the substantial computational advantage of surrogate prediction over repeated reference simulation.

The median-based serial screening cost combines ensemble evaluation with reference-simulation referrals. Offline training is treated separately in the break-even analysis, and final selected-design confirmation is outside this screening-cost calculation:

(24) $${\tilde{T}}_{\mathrm{closed}}=T_{\mathrm{ensemble}}+r{\tilde{T}}_{\mathrm{ref}},\;{\tilde{S}}_{\mathrm{closed}}=\frac{{\tilde{T}}_{\mathrm{ref}}}{{\tilde{T}}_{\mathrm{closed}}},$$

where $T_{\mathrm{ensemble}}=0.18549$ s is the estimated three-model evaluation time, *r* denotes the observed simulation-referral fraction, and ${\tilde{T}}_{\mathrm{ref}}=121.00$ s is the median reference-simulation time.

Under the 100 ms mean-delay budget, the low-observed-error global rule yields an estimated serial screening cost of 86.46 s per candidate, corresponding to a 1.40× speedup. The efficiency-oriented domain rule reduces the estimated cost to 25.33 s per candidate, achieving a 4.78× speedup with the 2.86% false-feasible fraction reported in the preceding section. This higher screening efficiency primarily results from the reduction in reference-simulation referrals.

These results demonstrate the practical benefit of integrating HSG-Net into the configuration exploration workflow. The surrogate rapidly evaluates candidate configurations, while selective reference simulation concentrates computational resources on cases requiring additional assessment. The two operating points provide different coverage–error trade-offs, allowing the screening strategy to be selected according to the evaluation requirements.

The mean training and evaluation time is 4754.42 s per model seed. With an existing labeled dataset, the computational savings from repeated candidate evaluation can offset the training cost of the three-model ensemble. The estimated break-even points are approximately 413 candidates for the low-observed-error rule and 150 candidates for the efficiency-oriented rule.

**Figure 8:**
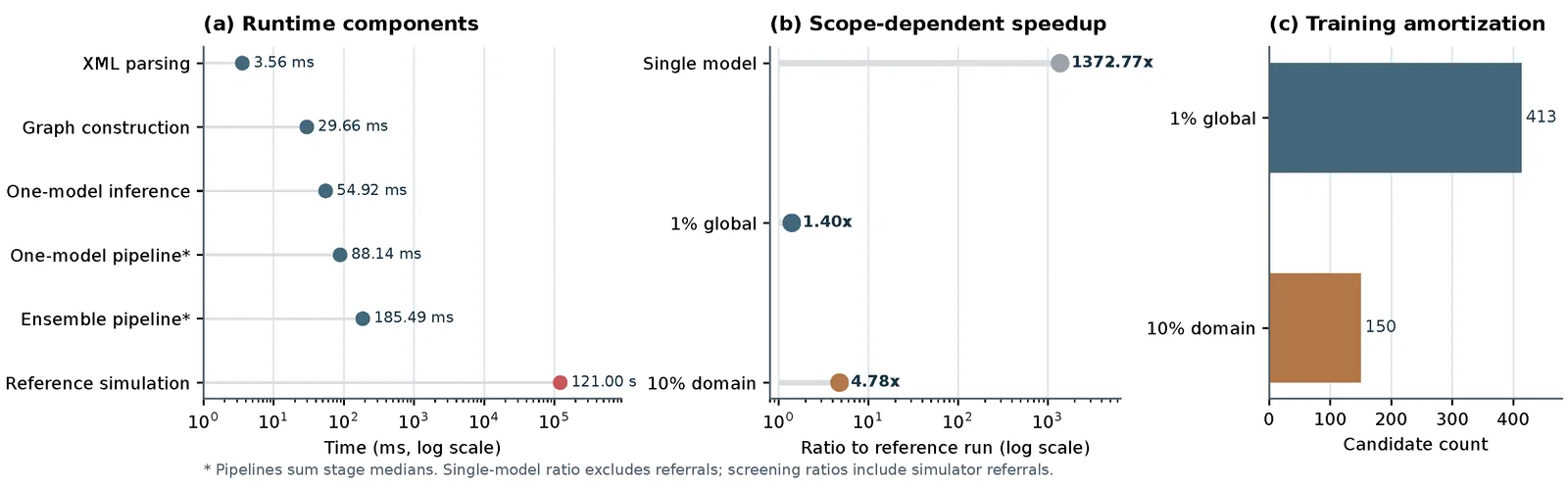
Computational efficiency of HSG-Net. (**a**) Runtime components and pipeline estimates, with reference-simulation time shown for comparison. (**b**) The isolated single-model pipeline ratio and median-based estimated serial screening speedups at two operating points. (**c**) Estimated three-model training break-even counts with an existing labeled corpus. The 1% global and 10% domain labels denote validation calibration targets, not test-set error rates.

Table 10 summarizes the measured component costs and estimated screening efficiency. Figure 8 presents the corresponding computational savings and training-cost amortization results.

## 5. Discussion

The results highlight the value of combining scheduling-mechanism descriptors with relational context for timing prediction. Flow-level descriptors characterize local timing conditions, while heterogeneous message passing represents interactions among tasks, partition service opportunities, and shared resources. Their combination achieves lower prediction error than the direct-flow-only alternative in the evaluated architecture comparison. The architecture balances predictive performance and computational cost: explicit role pooling is omitted because its removal preserves similar observed performance with fewer parameters, whereas relation factorization is retained based on its better validation performance and the negligible measured inference-time saving from further simplification. Scheduling relations explicitly represent task–partition dependencies, and the direct-flow pathway preserves local timing information alongside propagated context. These design choices reflect the combined evidence from validation performance, computational cost, and scheduling semantics. For offline exploration of aviation sensing-data processing chains, selective screening provides an additional trade-off between simulation effort and prediction errors. At the 100 ms budget, the low-observed-error operating point yields a median-based estimated serial speedup of 1.40×, with no false-feasible decisions observed among the 181 candidates predicted within budget. The efficiency-oriented point yields 4.78×, with 81 false-feasible decisions among 2835 such candidates. These exploratory operating points illustrate different allocations of simulation resources; the final selected designs still undergo reference simulation. The independent analytical comparisons support agreement for the evaluated scheduling mechanisms, and the controlled label-perturbation experiments demonstrate how reference-label bias can propagate into surrogate predictions. Together, these findings support scheduling-aware surrogate evaluation as a practical aid for repeated configuration assessment.

## 6. Conclusions

This paper presents HSG-Net for predicting mean end-to-end delay in partitioned aviation sensing-data processing chains. The method combines explicit scheduling descriptors, heterogeneous graph propagation, direct-flow preservation, and multi-scale decoding to represent interactions among task activation, partition windows, and shared resources. HSG-Net achieves an ensemble MAE of 6.372 ms, with 81.18% of test-flow predictions differing from the reference-simulation results by no more than 10 ms. Grouped testing, representative strict-domain holdouts, and architectural comparisons characterize its predictive performance across the evaluated configurations. An offline candidate-screening application further demonstrates distinct median-based estimated serial speedups of 1.40× and 4.78× at different screening-error operating points, excluding final selected-design confirmation. The proposed approach combines scheduling knowledge with learned relational representations to support accurate and efficient design-time evaluation. Future work will investigate transfer across broader configuration families and adaptive allocation of reference-simulation effort. 

## Figures and Tables

**Table 1:** Target-scope filtering and grouped data partition. Variability-filter counts refer to additional exclusions after applying the mean-delay bound. Base-scene counts describe the retained data.

Split	Labeled Flows	$\bar{d}>200$ ms	Variability Filter	Retained Flows	Base Scenes
Training	19,339	252	8	19,079	84
Validation	7578	494	67	7017	28
Test	7269	88	13	7168	26
Total	34,186	834	88	33,264	138

**Table 2:** Node inputs and associated relations in the HSG-Net heterogeneous graph. Flow inputs comprise 160 base features and 29 appended scheduling-mechanism descriptors.

Entity	Input Dim.	Main Information	Relations
Task	32	Execution-time bounds, activation type, configured period and deadline, priority, load, and slack	Message dependency, flow role, PIT assignment, core mapping, and contention
Flow	160+29	Path features, resource mapping, communication proxies, and scheduling-mechanism descriptors	Typed task–flow role relations and global context
PIT	12	Window offset, duration, end time, major-frame timing, and utilization	Task–PIT assignment, adjacent windows, and core mapping
Core	10	Local load, contention, and resource membership	Task/core, PIT/core, and CPU hierarchy
CPU	7	Resource identifiers, core and task counts, and aggregate partition-window statistics	Core–CPU and CPU–module relations
Module	6	Resource identifier, CPU and core counts, task counts, and aggregate partition-window duration	Resource hierarchy and context relations
Global	32	Configuration scale, task-load and timing statistics, partition utilization, and cross-resource dependency counts	Global-context relations

**Table 3:** Summary of reference-simulator evidence and label-sensitivity analysis.

Evidence Type	Evaluated Scope	Main Finding	Interpretation
Periodic analytical checks	Six isolated chains; 12 runs; 218 instances	Agreement at 0.001 ms export resolution	Agreement with analytical timing expectations for the evaluated periodic cases
Event/mixed analytical checks	Five cases; 358 instance-level outputs	Point/recurrence agreement and cross-window delays within the derived 35–36 ms completion-time range	Agreement with the evaluated event and mixed scheduling mechanisms
Execution repeatability	Eight configurations; six seeds; two repetitions	Identical-seed agreement; CV median 0.754%	Stable outputs for most evaluated pairs, with configuration-dependent variation
Observation-horizon sensitivity	Selected configurations; 1, 2, 5, and 10 s	Stable periodic case shows a 1.289% relative difference	Consistent mean-delay estimates under the evaluated stable periodic conditions
Label-error sensitivity	Clean, biased, and noisy-label training	Systematic label bias induces directional shifts in signed prediction error	Supervision-error propagation into surrogate predictions
Reproducibility evidence	Sanitized scenarios, analytical expectations, and recorded simulator comparisons	Case-level analytical and numerical evidence	Independent examination of selected scheduling cases and recorded results

**Table 4:** Multi-algorithm prediction performance on 7168 retained test flows. A10 is the fraction of flows with absolute error no larger than 10 ms.

Model	MAE (ms)	RMSE (ms)	$R^{2}$	SMAPE	A10
Ridge Regression	11.970	15.630	0.415	14.06%	52.99%
Histogram GBDT	12.429	17.131	0.297	14.55%	54.07%
Random Forest	11.803	15.710	0.409	13.86%	53.52%
Extra Trees	11.528	15.318	0.438	13.49%	54.48%
XGBoost	11.907	16.019	0.386	13.95%	53.64%
LightGBM	11.846	15.690	0.411	13.91%	53.60%
MLP ensemble	17.885	24.767	−0.469	20.67%	39.24%
GCN ensemble	11.949	15.491	0.425	14.03%	51.94%
GraphSAGE ensemble	11.869	15.365	0.435	13.99%	53.00%
GAT ensemble	11.958	15.545	0.421	14.08%	52.23%
HSG-Net (3 seeds)	6.799 ± 0.196	11.242 ± 0.105	0.697 ± 0.006	8.19% ± 0.15 pp	81.58% ± 1.48 pp
HSG-Net ensemble	6.372	10.426	0.740	7.71%	81.18%

Neural-model ensemble rows average predictions from three independently trained models. The HSG-Net three-seed row reports the run-wise mean with sample standard deviation below; pp denotes percentage points. Full neural-baseline seed statistics are reported in Table S5.

**Table 5:** HSG-Net ensemble performance by hardware domain in the grouped test set.

Domain	Flows	Scenes	MAE (ms)	$R^{2}$	A10
m2_c3	580	2	7.659	0.479	68.62%
m2_c4	996	2	5.846	0.775	86.45%
m3_c4	983	4	6.496	0.745	82.40%
m3_c5	1156	4	7.240	0.704	71.28%
m4_c4	425	1	7.346	0.662	84.00%
m4_c5	409	2	5.205	0.516	83.37%
m4_c6	934	2	5.049	0.832	85.76%
m4_c7	979	3	5.688	0.751	86.82%
m4_c8	37	1	18.249	−4.662	27.03%
m5_c6	215	1	6.603	0.783	85.58%
m5_c7	44	1	10.320	0.319	79.55%
m5_c8	97	1	5.696	−2.751	92.78%
m5_c9	181	1	7.367	−0.116	76.80%
m5_c10	132	1	5.167	−0.269	90.15%

**Table 6:** Strict leave-one-domain-out generalization. Each method is an ensemble of three independently seeded models. Parentheses indicate base scenes/flows; confidence intervals use grouped scene bootstrap. Bold indicates the best performance among the compared methods within each target domain.

Domain (Scenes/Flows)	Model	Seed MAE (ms)	Ensemble MAE [95% CI] (ms)	A10
m2_c3 (16/4267)	HSG-Net	7.994 ± 0.157	**7.485 [4.220, 11.354]**	**78.84%**
	XGBoost	13.106 ± 0.012	13.102 [8.230, 19.544]	55.31%
	GraphSAGE	12.763 ± 0.153	12.707 [8.245, 18.487]	56.60%
m3_c5 (21/6102)	HSG-Net	8.334 ± 0.205	**7.842 [6.490, 9.148]**	**76.57%**
	XGBoost	13.940 ± 0.069	13.936 [11.593, 15.711]	43.23%
	GraphSAGE	13.810 ± 0.095	13.727 [11.356, 15.462]	43.95%
m4_c8 (2/583)	HSG-Net	6.901 ± 0.776	**6.474 [5.683, 18.144]**	**85.08%**
	XGBoost	11.317 ± 0.011	11.317 [10.493, 23.472]	54.72%
	GraphSAGE	11.438 ± 0.391	11.380 [10.595, 22.962]	54.72%
m5_c7 (7/469)	HSG-Net	9.090 ± 0.966	**8.389 [6.046, 8.760]**	**77.61%**
	XGBoost	16.563 ± 0.178	16.543 [9.323, 17.981]	37.74%
	GraphSAGE	15.868 ± 0.137	15.819 [8.049, 17.500]	39.87%

**Table 7:** Architecture comparison under the common training protocol. Validation MAE is the mean best value across three seeds; test MAE and A10 use three-seed ensemble predictions. Inference time is measured for a single model.

Architecture	Val. MAE	Test MAE	A10	Params.	Inference
	(ms)	(ms)	(%)	(M)	(ms)
HSG-Net (Flow-node readout)	8.445	6.372	81.18	2.687	54.92
With explicit role pooling	8.375	6.417	82.00	3.146	57.20
Unified heterogeneous propagation	9.175	6.528	81.85	2.214	54.86
Direct-flow-only	11.931	9.953	63.35	0.138	2.21

**Table 8:** Matched module deletions relative to the role-pooling-free HSG-Net. MAE and A10 use three-seed ensemble predictions. ΔMAE is the original-sample difference between each variant and HSG-Net; intervals use 10,000 paired base-scene bootstrap resamples.

Configuration	MAE (ms)	A10 (%)	ΔMAE (ms)	95% Interval (ms)
HSG-Net	6.372	81.18	—	—
w/o relation factorization	6.528	81.85	+0.156	[−0.172, 0.453]
w/o scheduling relations	6.452	81.56	+0.080	[−0.196, 0.341]
w/o direct-flow preservation	6.599	81.05	+0.228	[−0.033, 0.504]
w/o scheduling relations and direct-flow preservation	6.625	81.00	+0.253	[−0.061, 0.543]

**Table 9:** Offline candidate screening under a 100 ms mean-delay budget. Automatic coverage includes both within-budget and over-budget decisions. False-feasible fractions are calculated among candidates automatically classified as within budget.

Metric	Low-Observed-Error Global Rule	Efficiency-Oriented Domain Rule
Validation empirical target	1%	10%
Predicted within budget	181	2835
Predicted over budget	1327	1327
Referred to simulation	3746	1092
Automatic coverage	28.70%	79.22%
False-feasible decisions	0/181	81/2835
False-feasible fraction	0.00%	2.86%
Fraction of true violations predicted feasible	0.00%	6.52%
False over-budget decisions	264	264

**Table 10:** Measured computational components and estimated serial screening efficiency. Pipeline costs combine model inference, XML parsing, and graph construction; referral-adjusted costs additionally account for reference-simulation referrals.

Measurement and Scope	Value
Mean training/evaluation time per seed	4754.42 s
Checkpoint size: one model/ensemble	10.49/31.46 MiB
Warm-process model cold load	210.29 ms
Existing XML parsing/graph construction	3.56/29.66 ms
Warm inference including tensor transfer: one/ensemble	54.92/152.26 ms
Stage-sum single-model pipeline estimate	88.14 ms
Existing median reference run	121.00 s
Isolated component ratio (estimated)	1372.77×
Batch-64 device-resident throughput: one/ensemble	1449.68/471.67 graphs/s
Low-observed-error 100-ms serial cost/speedup (estimated)	86.46 s/1.40×
Low-observed-error ensemble training amortization (existing corpus)	413 candidates
Efficiency-oriented 100-ms serial cost/speedup (estimated)	25.33 s/4.78×
Efficiency-oriented ensemble training amortization (existing corpus)	150 candidates

## Data Availability

De-identified scenario descriptions, analytical timing expectations, recorded simulator comparisons, and execution-repeatability statistics are provided in the Supplementary Material, together with the numerical results supporting the prediction and screening analyses. These materials support case-level assessment and partial reproducibility of the reported analyses. The reference-simulator source code, production configurations, and engineering acceptance materials are not publicly available because of project confidentiality restrictions.

## Supplementary Materials

The following supporting information can be downloaded at https://www.mdpi.com/article/10.3390/s26206662/s1. The Supplementary Material contains the following sections: Static Mechanism Descriptor Provenance (Section S1); Directed Relation Types and Final Architecture (Section S2); Model Inputs and Training Configurations (Section S3); Audited Data and Evaluation Scope (Section S4); Three-Seed Stability of Neural Baselines and HSG-Net (Section S5); Ensemble Dependence (Section S6); Screening and Computational Cost (Section S7); Execution Repeatability (Section S8); Independent Periodic Scheduling Cases (Section S9); Paired Effects and Multiplicity (Section S10); Observation-Horizon Audit (Section S11); Controlled Label-Error Propagation (Section S12); Event-Triggered and Mixed-Activation Cases (Section S13); Final-Model Ablation Settings and Detailed Results (Section S14); and Architecture Validation and Complexity Measurements (Section S15).

## Abbreviations

The following abbreviations are used in this manuscript:

ARINC	Aeronautical Radio, Incorporated
CPU	Central Processing Unit
GAT	Graph Attention Network
GCN	Graph Convolutional Network
GBDT	Gradient Boosting Decision Tree
GNN	Graph Neural Network
GPU	Graphics Processing Unit
HSG-Net	Scheduling-Aware Heterogeneous Graph Surrogate
MAE	Mean Absolute Error
MLP	Multilayer Perceptron
PIT	Partition Information Table
RMSE	Root Mean Squared Error
SMAPE	Symmetric Mean Absolute Percentage Error
XML	Extensible Markup Language
