# Supplementary material for: HSG-Net: A Scheduling-Aware Heterogeneous Graph Surrogate for Aviation Sensing-Data Processing Chains
Source: Sensors (Basel). 2026 Oct 21;26(20):6662. doi: 10.3390/s26206662 (PMC13709372; doi:10.3390/s26206662)
Supplement: Supplementary file 1 [file sensors-26-06662-s001.zip › sensors-4597354-supplementary.pdf]

Supplementary Material

This supplement provides feature and relation definitions, training settings, seed-level results, and detailed statistical and analytical evidence supporting the manuscript.

S1. Static Mechanism Descriptor Provenance

All descriptors are computed from candidate XML configurations before inference. None uses reference delays, completed-instance counts, or simulator trace statistics. Each Flow node receives 160 base features and the 29 descriptors in Table S1.

Table S1. Static mechanism descriptors

ID	Descriptor	Unit	Definition
F01	path_exec_sum	ms	Sum of configured maximum execution times on the flow path.
F02	path_min_exec_sum	ms	Sum of configured minimum execution times on the flow path.
F03	path_period_min	ms	Minimum configured task period on the path.
F04	path_period_max	ms	Maximum configured task period on the path.
F05	path_period_ratio	ratio	Ratio of maximum to minimum path period.
F06	path_slack_min	ms	Minimum configured deadline-minus-execution slack on the path.
F07	cross_module	binary	Indicator that the flow crosses a module boundary.
F08	cross_cpu	binary	Indicator that the flow crosses a CPU boundary.
F09	logic_message_period_sum	ms	Sum of configured message periods along the path.
F10	logic_comm_delay_ms_proxy_sum	ms	Sum of static serialization and mapping-class transfer-delay proxies.
F11	logic_comm_delay_to_dst_period_sum	ratio	Sum of transfer-delay proxy divided by each destination period.
F12	logic_dst_exec_to_period_sum	ratio	Sum of destination maximum execution time divided by destination period.
F13	logic_dst_exec_to_pit_duration_sum	ratio	Sum of destination maximum execution time divided by PIT-window duration.
F14	logic_dst_window_exec_slack_min	ms	Minimum PIT duration minus destination maximum execution time.
F15	logic_dst_priority_mean	priority	Mean configured priority of downstream tasks.
F16	logic_same_core_share	share	Share of path edges whose endpoint tasks use the same core.
F17	logic_dst_same_pit_competition_exec_to_window_max	ratio	Maximum same-PIT competing execution demand divided by PIT duration.

ID	Descriptor	Unit	Definition
F18	logic_arrival_phase_ratio_sum	ratio	Sum of estimated arrival phase divided by destination period.
F19	logic_arrival_phase_ratio_max	ratio	Maximum estimated arrival phase divided by destination period.
F20	logic_arrival_before_dst_window_share	share	Share of edges whose latest estimated arrival precedes the PIT window.
F21	logic_arrival_inside_dst_window_share	share	Share of edges whose latest estimated arrival lies inside the PIT window.
F22	logic_arrival_min_after_dst_offset_share	share	Share of edges whose earliest estimated arrival is after the PIT offset.
F23	logic_net_delay_flips_dst_offset_share	share	Share for which the transfer proxy moves arrival across the PIT start.
F24	logic_net_delay_flips_dst_window_share	share	Share for which the transfer proxy moves arrival across the PIT end.
F25	logic_periodic_wait_ms_proxy_sum	ms	Sum of analytically estimated periodic-waiting proxies.
F26	logic_periodic_wait_ms_proxy_max	ms	Maximum analytically estimated periodic-waiting proxy.
F27	logic_dst_start_miss_wait_ms_proxy_sum	ms	Sum of periodic start-boundary waiting proxies.
F28	logic_partition_window_miss_wait_ms_proxy_sum	ms	Sum of partition-window-end waiting proxies.
F29	logic_src_finish_to_dst_offset_gap_min	ms	Minimum destination PIT offset minus estimated source completion time.

S2. Directed Relation Types and Final Architecture

The graphs contain 35 directed relation types, with explicit reverse relations where applicable. The final HSG-Net reads the propagated Flow-node state directly and retains typed task–flow role edges. Graph and direct-flow prediction heads produce output tuples combined component-wise before multi-scale decoding: o_f = gamma_f o_G + (1 - gamma_f)o_0. Each model has 2,687,055 parameters.

Table S2. Directed relations

Family	Forward relation	Reverse/companion
Dependency	Task–sends_to–Task	Task–rev_sends_to–Task
Resource mapping	Task–runs_on–Core	Core–rev_runs_on–Task
Resource hierarchy	Core–belongs_to–CPU	CPU–rev_belongs_to_core–Core
Resource hierarchy	CPU–belongs_to–Module	Module–rev_belongs_to_cpu–CPU
Flow membership	Task–part_of–Flow	Flow–rev_part_of–Task

Family	Forward relation	Reverse/companion
Flow role	Task–source_of–Flow	Flow–rev_source_of–Task
Flow role	Task–middle_of–Flow	Flow–rev_middle_of–Task
Flow role	Task–sink_of–Flow	Flow–rev_sink_of–Task
Partition scheduling	Task–scheduled_in–PIT	PIT–rev_scheduled_in–Task
Contention	Task–competes_with–Task	Direction stored explicitly
PIT sequence	PIT–next_pit–PIT	PIT–prev_pit–PIT
PIT mapping	PIT–maps_to–Core	Core–rev_maps_to–PIT
Global context	Global–context_to_task–Task	Task–rev_context_to_task–Global
Global context	Global–context_to_flow–Flow	Flow–rev_context_to_flow–Global
Global context	Global–context_to_pit–PIT	PIT–rev_context_to_pit–Global
Global context	Global–context_to_core–Core	Core–rev_context_to_core–Global
Global context	Global–context_to_cpu–CPU	CPU–rev_context_to_cpu–Global
Global context	Global–context_to_module–Module	Module–rev_context_to_module–Global

S3 Model Inputs and Training Configurations

S3.1 Input Coverage and Representation

This section describes the matched inputs used in the baseline comparisons and the input configuration of HSG-Net without role pooling.

Group I comprises the actual model-input attributes of seven entity types and the Flow period unit; Group II comprises F01–F29 in Table S1; Group III comprises 35 directed relations and their endpoints; Group IV comprises relation-specific edge attributes.

Table S3a Input coverage and representation

Item	Tabular regressors and MLP	GCN, GraphSAGE and GAT ensembles	HSG-Net ensemble
Entity attributes I	All seven entity types and Flow period units	Same node fields; period unit supplied to the Flow prediction head	Seven entity types; separate Flow period-unit input
Descriptors II	F01–F29 for every Flow	F01–F29 within 189-dimensional Flow features	160 base + 29 scheduling features = 189

Item	Tabular regressors and MLP	GCN, GraphSAGE and GAT ensembles	HSG-Net ensemble
Relations III	35 blocks retaining edge direction and local endpoints	Original 35 directed relations; no relation nodes	Native 35 directed heterogeneous relations
Edge attributes IV	Original attributes retained per edge: 0/12/16/30 dimensions	95-dimensional input: type one-hot, attributes and validity mask; encoded and aggregated to nodes	Native relation-specific edge attributes
Dimensions	15,900 total, including 194 target-specific dimensions	Node dimensions as in Table S3c; period side input: 1; edge adapter input: 95; encoding and propagation: 96	Node dimensions as in Table S3c; period side input: 1; encoding and propagation: 112
Organization	Original-order flattening, zero padding and validity indicators; no truncation	Common edge-information adapter; two original-graph propagation layers; target-Flow readout and period side input	Two propagation layers; direct-Flow pathway; no role pooling

Tabular models comprise Ridge Regression, Histogram GBDT, Random Forest, Extra Trees, XGBoost, LightGBM and the MLP ensemble. They share the same input vector.

Input Encoding Details

Tabular serialization

The configuration representation has 15,706 dimensions: 2,818 node-attribute values + 98 node-validity indicators + 2,898 edge-validity and endpoint entries + 9,888 edge-attribute values + 4 Flow period units. The target-specific input has 194 dimensions: 189 target-Flow attributes + 1 period unit + a 4-dimensional selection mask. The total is 15,900; the 194 dimensions are not appended a second time.

Entities and edges retain their original graph order. Fixed slots use zero padding and validity indicators, without statistical aggregation or truncation. Capacities come from an allocation inventory of existing input shapes and use structural sizes rather than labels. Exceeding any capacity raises an error and requires uniform expansion and rebuilding before training; excess entities must not be discarded. Tables S3c–S3d give the capacities.

The four-dimensional target mask supports configurations with at most four Flows under this encoding. Entry $j = 1$ selects original local Flow index j; all other entries are zero. Unused slots remain zero and are distinguished by node-validity indicators. This is not a global Flow identifier and does not constrain path length. Supporting more Flows requires capacity expansion and changes the input dimension D.

Common graph-baseline adapter

The adapter retains all original nodes and edges. A type-specific linear encoder maps each node to a 96-dimensional vector h. For each edge, b concatenates a 35-dimensional relation-type one-hot vector, original attributes padded to 30 dimensions, and a 30-dimensional attribute-validity mask: $\dim(b) = 35 + 30 + 30 = 95$. A shared linear encoder maps b to a 96-dimensional vector e. For relations without numerical attributes, attribute values and validity masks are zero, while the relation-type one-hot vector remains present.

For each node, incoming and outgoing edge vectors e are averaged separately. Concatenating h, the incoming mean and the outgoing mean gives 288 dimensions, which a linear layer maps to 96 dimensions. The original GCN, GraphSAGE or GAT operator then performs two propagation layers with hidden width 96. All three baselines share this adapter. Target-Flow readout is concatenated with its one-dimensional period side input and passed to a common prediction head.

This is learned encoding and aggregation of common raw information, not a claim that individual edge attributes can be reconstructed losslessly from the aggregate. Lossless preservation refers only to the stored original graph input and tabular serialization. The graph baselines use this common input adapter. Matching prediction inputs does not imply

identical inductive biases or training objectives; HSG-Net task-level auxiliary supervision is described separately. Any newly fitted preprocessing statistics use only the training split.

S3.2 Training Configurations

The verified HSG-Net configuration uses 112-dimensional node encodings and two propagation layers with hidden width 112. Its raw Flow input is 189-dimensional; the period unit is a separate one-dimensional side input, not a 190th node feature. The graph baselines use 96-dimensional encodings and two propagation layers with hidden width 96. Input matching does not require equal hidden widths.

Table S3b lists the training hyperparameters.

Table S3b Training settings

Method	Training settings
Ridge	alpha = 3.0; median imputation; standard scaling.
Histogram GBDT	320 iterations; learning rate 0.04; 31 leaves; minimum leaf size 20.
Random Forest	420 trees; feature fraction 0.8; minimum leaf size 2.
Extra Trees	420 trees; feature fraction 0.9; minimum leaf size 2.
XGBoost	220 trees; depth 5; learning rate 0.04; row/column fractions 0.86/0.82.
LightGBM	420 trees; 31 leaves; learning rate 0.035; row/column fractions 0.88/0.85.
MLP	Hidden widths 96/48; Adam; learning rate 0.0005; batch size 256; validation-based early stopping.
GCN/GraphSAGE/GAT	Two layers; hidden size 96; dropout 0.16; batch size 128; AdamW learning rate 0.0008; validation-based early stopping.
HSG-Net	Two layers; hidden size 112; four attention heads; dropout 0.12; batch size 96; AdamW learning rate 0.00045; weight decay 0.00015; maximum 36 epochs; patience 12; validation-MAE checkpoint selection.

HSG-Net uses three seeds and validation-MAE checkpoint selection. Ensemble predictions are averaged before metrics are calculated. Paired intervals use 10,000 complete-base-scene resamples, with original-sample effects and bootstrap means reported separately.

S3.3 Serialization Dimensions and Capacities

Table S3c Entity input dimensions and fixed slot capacities

Entity type	Attribute dimensions	Slot capacity
task	32	51
flow	189	4
pit	12	16

Entity type	Attribute dimensions	Slot capacity
core	10	10
cpu	7	10
module	6	6
global	32	1

The entity order is Task, Flow, PIT, Core, CPU, Module and Global. Attribute dimensions are 32/189/12/10/7/6/32, respectively, with 98 node slots and 2,818 attribute entries in total. Core nodes correspond only to distinct Module–CPU–Core combinations appearing in task mappings; physical cores without mapped tasks are not represented as separate Core nodes. Thus, the 16–80 cores reported in the manuscript refer to the total physical-core count, whereas the capacity of ten Core nodes in Table S3c refers to the graph representation. Serialization preserves all nodes and relations in the model graph without truncation. Flow period units are stored separately.

Message-dependency edges have 30 attributes; Flow membership/role, task–PIT scheduling and task-contention edges have 16; PIT-sequence edges have 12; other relations have no numerical edge attributes. The 35 relation types allocate 966 edge slots and 9,888 attribute entries. Each edge slot also stores one validity indicator and two endpoint indices, giving 966 × 3 = 2,898 entries.

Table S3d Edge slot capacities by directed relation

Relation (source | type | target)	Slot capacity	Attribute dimensions
task|sends_to|task	16	30
task|rev_sends_to|task	16	30
task|runs_on|core	51	0
core|rev_runs_on|task	51	0
pit|maps_to|core	16	0
core|rev_maps_to|pit	16	0
core|belongs_to|cpu	10	0
cpu|rev_belongs_to_core|core	10	0
cpu|belongs_to|module	10	0
module|rev_belongs_to_cpu|cpu	10	0
task|part_of|flow	20	16
flow|rev_part_of|task	20	16

Relation (source | type | target)	Slot capacity	Attribute dimensions
task|source_of|flow	4	16
flow|rev_source_of|task	4	16
task|middle_of|flow	24	16
flow|rev_middle_of|task	24	16
task|sink_of|flow	4	16
flow|rev_sink_of|task	4	16
global|context_to_task|task	51	0
task|rev_context_to_task|global	51	0
global|context_to_flow|flow	4	0
flow|rev_context_to_flow|global	4	0
global|context_to_pit|pit	16	0
pit|rev_context_to_pit|global	16	0
global|context_to_core|core	10	0
core|rev_context_to_core|global	10	0
global|context_to_cpu|cpu	10	0
cpu|rev_context_to_cpu|global	10	0
global|context_to_module|module	6	0
module|rev_context_to_module|global	6	0
task|scheduled_in|pit	51	16
pit|rev_scheduled_in|task	51	16
task|competes_with|task	328	16
pit|next_pit|pit	16	12
pit|prev_pit|pit	16	12

S4. Audited Data and Evaluation Scope

Table S4. Data and evaluation scope

Item	Value
XML configurations	24,516
Flow labels before filtering	34,186
Retained flows	33,264
Retained configurations	23,716
Base scenes	138
Training / validation / test scenes	84 / 28 / 26
Observation horizon / executions per XML	1 s / 1
Completed instances per flow	1–48; median 19
Neural seeds	20260903, 20260913, 20260923
Bootstrap unit / repetitions	Base scene; 10,000
Screening budget	100 ms
Calibration / evaluation scenes	28 / 26
Calibration / evaluation candidates	4732 / 5254

The legacy XML corpus does not store an explicit simulator random seed. Each XML provides a fixed-horizon reference evaluation. Neural seeds and split identifiers are retained. Scenario descriptions, analytical comparisons, and recorded statistics support partial reproducibility; confidential source code, production XML, and engineering acceptance materials are not included.

S5. Three-Seed Stability of Neural Baselines and HSG-Net

Table S5. Seed stability on 7168 retained test flows

Model	MAE mean ± SD (ms)	Ensemble MAE (ms)	A10 mean ± SD (%)	Ensemble A10 (%)
MLP	20.874 ± 2.303	17.885	35.87 ± 1.69	39.24
GCN	12.031 ± 0.159	11.949	51.83 ± 0.59	51.94

Model	MAE mean ± SD (ms)	Ensemble MAE (ms)	A10 mean ± SD (%)	Ensemble A10 (%)
GraphSAGE	11.931 ± 0.115	11.869	52.48 ± 1.15	53.00
GAT	12.022 ± 0.028	11.958	52.16 ± 0.89	52.23
HSG-Net	6.799 ± 0.196	6.372	81.58 ± 1.48	81.18

The final HSG-Net ensemble has RMSE 10.426 ms, R2 0.7397, and SMAPE 7.705%.

S6. Ensemble Dependence

Three independently seeded models are evaluated using pairwise Pearson correlations of predictions and residuals. The correlations are 0.933–0.953 and 0.776–0.832, respectively. Averaging predictions reduces MAE from the member-average 6.799 ms to 6.372 ms. The Spearman correlation between member disagreement and ensemble absolute error is 0.299.

seed_a	seed_b	prediction_pearson	residual_pearson
20260903	20260913	0.933	0.776
20260903	20260923	0.940	0.781
20260913	20260923	0.953	0.832

Interpretation

High prediction correlation is expected when members learn the same targets from the same data. Residual correlation more directly describes shared prediction errors. The MAE reduction from 6.799 to 6.372 ms shows that averaging nevertheless improves this metric, because member errors are not identical. The disagreement–error correlation of 0.299 motivates treating disagreement as an empirical association rather than using it as the screening rule; candidate screening instead uses the validation-calibrated mean candidate score.

S7. Screening and Computational Cost

The supplementary operating-point comparison uses a 100 ms reference mean-delay budget. The calibration target alpha determines the eligible empirical false-feasible fraction on validation candidates; it is distinct from the prediction threshold. Domain-conditional calibration uses domains with at least 30 validation candidates and otherwise falls back to the global threshold at the same alpha.

False-feasible fraction is the number of reference-budget violations predicted within budget divided by all candidates predicted within budget. Automatic coverage includes both predicted-within-budget and predicted-over-budget decisions. The manuscript reports the global alpha = 0.01 and domain alpha = 0.10 points; the table additionally lists alternative operating points.

Serial screening cost is estimated as 0.185487275 s + r × 121 s, where r is the simulation-referral fraction. The reference-runtime median is 121 s. Final selected-design confirmation and corpus generation are outside this calculation. The single-model pipeline stage-median sum is 88.1431 ms; transfer-inclusive inference medians are 54.9193 ms for one model and 152.263475 ms for the ensemble.

Policy	α	Coverage (%)	False feasible / predicted feasible	Conditional proportion (%)	Estimated serial speedup
Global	0.01	28.702	0 / 181	0	1.4
Domain	0.01	48.477	27 / 1220	2.213	1.935
Global	0.1	90.826	110 / 3445	3.193	10.721
Domain	0.1	79.216	81 / 2835	2.857	4.776

Interpretation of the operating points

At the global 1% calibration target, 0 of 181 predicted-within-budget candidates are false-feasible and automatic coverage is 28.70%; most candidates therefore still require simulation. At the domain 10% target, coverage rises to 79.22%, with 81 of 2835 predicted-within-budget candidates exceeding the reference budget. The referral reduction explains the change from 1.40x to 4.78x estimated serial speedup. The validation targets label calibration policies, not achieved test error rates. Final selected designs are confirmed by reference simulation, so the measured trade-off concerns preliminary candidate selection and simulation allocation.

S8. Execution Repeatability

Eight configurations are evaluated with six seeds and two repetitions per seed, yielding 96 executions and 23 scenario–flow pairs. Repeated executions with the same seed have identical recorded outputs. Between-seed summaries use six seed means per pair.

The approximate 95% interval is the mean plus or minus t(0.975,5) times the sample standard deviation divided by the square root of six. It describes execution variability. The table also records absolute differences from historical labels for 13 matched retained flows; nine historical labels fall inside the approximate single-run prediction interval.

Quantity	Median	P90	Maximum
Between-seed CV (%)	0.754	4.02	9.615
Approximate 95% seed-mean interval width (ms)	0.861	5.926	19.889
Absolute historical-label shift, 13 matched retained flows (ms)	1.677	14.064	46.033

Interpretation

Same-seed equality establishes that repeated execution reproduces the recorded statistics when configuration and random conditions are fixed. Between-seed CV measures a different property: sensitivity to the tested execution randomness. The median CV of 0.754% and P90 of 4.020% indicate relatively small variability for most evaluated pairs, while the maximum of 9.615% identifies configuration-dependent variation. The seed-mean intervals quantify this repeatability evidence rather than discrepancy from physical measurements.

S9. Independent Periodic Scheduling Cases

Six isolated three-task periodic chains use fixed 2 ms execution times and predefined partition service opportunities. The analytical recurrence determines each downstream eligible opportunity from predecessor completion and the declared transfer interval 0 < tau < 1 ms, independently of simulator code and outputs.

The predeclared acceptance tolerance is 0.3 ms and the output resolution is 0.001 ms. Across two seeds, 12 runs and 218 completed flow-instance comparisons, the maximum reported exported difference is 0.000 ms. The table gives the case parameters, analytical expectations and recorded outputs.

Case	Period (ms)	Offsets (ms)	Analytical delay (ms)	Simulator delay (ms)	Maximum difference (ms)
Current opportunities	25	0 / 5 / 10	12	12	0.000
Missed middle opportunity	25	0 / 1 / 10	37	37	0.000
Missed sink opportunity	25	0 / 5 / 6	33	33	0.000
Changed major frame	40	0 / 7 / 14	16	16	0.000
Delayed source phase	25	4 / 10 / 16	14	14	0.000
Two missed opportunities	25	4 / 3 / 2	50	50	0.000

Analytical construction

The periodic checks use a three-task chain with a common period P, task service offsets a1, a2 and a3, and execution time e = 2 ms. For the first source instance, s1 = a1 and c1 = s1 + e. For each downstream task i, select the earliest nonnegative integer k_i such that s_i = a_i + k_i P is no earlier than c_(i-1) + tau_(i-1); then c_i = s_i + e. The analytical flow delay is c3 - s1. The assumed link transfers satisfy 0 < tau < 1 ms. This recurrence is specified from the case parameters, rather than fitted to recorded delays.

For these isolated cases, the source releases repeat with the same phase. Applying the recurrence to each completed instance yields the same expected delay for that case. Comparing instance-level outputs therefore tests the repeated timing behavior, not merely agreement between two aggregate means.

Case	Parameters (ms)	Task start → completion (ms)	Delay (ms)	Interpretation
Current service opportunities	P=25, offsets=(0,5,10)	0→2; 5→7; 10→12	12−0=12	Each predecessor finishes early enough for its successor to use the same-cycle opportunity.
Missed middle opportunity	P=25, offsets=(0,1,10)	0→2; 26→28; 35→37	37−0=37	The middle opportunity at 1 ms precedes message availability. Its next start is 26 ms; the sink then uses 35 ms.
Missed sink opportunity	P=25, offsets=(0,5,6)	0→2; 5→7; 31→33	33−0=33	The middle task finishes at 7 ms, after the sink opportunity at 6 ms; the next sink opportunity is 31 ms.
Changed period	P=40, offsets=(0,7,14)	0→2; 7→9; 14→16	16−0=16	The chain completes within the current cycle under the changed period and offsets.

Case	Parameters (ms)	Task start → completion (ms)	Delay (ms)	Interpretation
Delayed source phase	P=25, offsets=(4,10,16)	4→6; 10→12; 16→18	18−4=14	Delay is measured from source execution, not from time zero.
Two missed opportunities	P=25, offsets=(4,3,2)	4→6; 28→30; 52→54	54−4=50	The middle and sink each wait for a later eligible opportunity, creating cumulative phase waiting.

Interpretation of the comparisons

The six cases distinguish same-cycle propagation, individual missed opportunities, accumulated waiting and a nonzero source phase. Agreement across the 218 completed instances supports the simulator's reproduction of these evaluated periodic service-opportunity mechanisms. The two seeds check repeatability of the same six analytical cases; they do not constitute additional analytical models.

S10. Paired Effects and Multiplicity

Paired MAE differences are calculated on the original test predictions. Confidence intervals use 10,000 paired resamples of the 26 complete base scenes. Bootstrap means are reported separately from original-sample differences.

The scene-level sign-flip analysis and Holm-adjusted probabilities supplement the paired intervals. These statistics describe component effects for the fitted models on the evaluated scenes.

comparison	original_delta_mae_ms	bootstrap_mean_delta_ms	ci95_low_ms	ci95_high_ms	repeats
role_pooling_added_minus_final	0.046	0.042	-0.242	0.282	10000
no_factorization_minus_final	0.156	0.157	-0.172	0.453	10000
direct_only_minus_final	3.581	3.556	2.404	4.740	10000
no_scheduling_minus_final	0.080	0.075	-0.196	0.341	10000
no_direct_minus_final	0.228	0.229	-0.033	0.504	10000
no_scheduling_direct_minus_final	0.253	0.249	-0.061	0.543	10000

variant	original_delta_mae_ms	scene_sign_flip_p	holm_adjusted_p	sign_flip_repeats
no_factorization	0.156	0.370	0.740	100000
no_scheduling	0.080	0.588	0.740	100000
no_direct	0.228	0.111	0.332	100000

S11. Observation-Horizon Audit

Two configurations—a stable periodic configuration and a high-delay/variability configuration—are evaluated at observation horizons of 1, 2, 5, and 10 s, yielding eight configuration–horizon combinations. The simulator seed is fixed, and all settings other than the observation horizon are unchanged. Each mean is calculated over flow instances completed within the corresponding horizon.

The table summarizes the 1 s and 10 s results for both configurations. The reported maximum relative changes are 1.289% for the stable periodic configuration and 11.085% for the high-delay/variability configuration.

Configuration category	Flows	1 s mean range (ms)	10 s mean range (ms)	Maximum relative change (%)
Stable periodic	4	29.135–57.732	29.515–57.306	1.289
High delay / variability	4	27.235–117.233	26.659–131.849	11.085

Interpretation

Stable periodic cases repeat the same phase alignment, allowing additional observation cycles to yield similar completed-instance averages. By contrast, a mean calculated only over completed instances can change as a longer horizon includes later completions with different waiting histories. The four-category comparison therefore supports reporting the observation horizon as part of the label definition rather than treating all finite-horizon means as interchangeable.

S12. Controlled Label-Error Propagation

Training and validation mean-delay labels receive a -5% systematic bias, a +5% systematic bias, or one fixed 5% zero-mean random-noise realization. Each condition uses three seeds and the same training and checkpoint-selection protocol. Nominal test labels are unchanged. The clean condition uses the final role-pooling-free HSG-Net.

Signed error is prediction minus reference. The table reports prediction metrics, original-sample MAE changes, bootstrap means and paired 95% intervals from 10,000 base-scene resamples. The opposite signed-error shifts under negative and positive label bias demonstrate controlled supervision-error propagation.

model	mae_ms	rmse_ms	r2	a10_percent	signed_error_ms	original_delta_mae_ms	bootstrap_mean_delta_ms	ci95_low_ms	ci95_high_ms
clean	6.372	10.426	0.740	81.180	0.417	—	—	—	—
minus5	7.535	11.107	0.705	79.646	-4.791	1.164	1.148	0.490	1.748
plus5	7.781	11.273	0.696	76.897	3.496	1.410	1.396	0.799	1.849
noise5	6.710	10.897	0.716	81.013	-1.632	0.339	0.330	-0.070	0.689

Interpretation of controlled retraining

Keeping nominal test labels fixed gives all retrained models the same evaluation reference. Negative-bias training shifts signed error by -5.208 ms relative to clean, while positive-bias training shifts it by +3.079 ms. These opposite shifts connect supervision direction with prediction direction, providing experimental evidence for propagation rather than merely asserting that a surrogate learns its labels.

Negative-bias training increases MAE by 1.164 ms with a paired interval wholly above zero. Positive-bias training increases MAE by 1.410 ms. The noise realization increases MAE by 0.339 ms with interval [-0.070,0.689] ms. Thus systematic perturbations produce larger aggregate effects in these comparisons, whereas the tested noise realization gives a smaller, less clearly separated change. The applied perturbations are sensitivity scenarios, not measurements of actual simulator bias.

S13. Event-Triggered and Mixed-Activation Cases

Five cases evaluate event-triggered processing, capture of an upcoming partition window, execution carried over to the next partition window, a minimum inter-arrival constraint and mixed periodic/event activation. Analytical expectations are derived from the case inputs and scheduling assumptions independently of simulator outputs.

Each seed produces 179 completed flow-instance outputs: 358 observations across ten runs. Four point-value or recurrence cases agree at the exported resolution. For the cross-window case, the timing analysis accounts for partial execution before window closure and resumption of the remaining execution in the next window. Under the assumptions detailed below, the completion-time relation is t_finish = 25 + a ms, where a is the terminal-task start time expressed in milliseconds relative to the corresponding source start. For a in [10,11] ms, this gives a deterministic delay range of [35,36] ms, containing the recorded delays of 35.500–36.000 ms. The capture case places arrival before window opening. A 2 ms offset change produces a recorded delay increase of 24.5–25 ms.

Case	Objective / scenario	Instances per seed	Analytical expectation (ms)	Simulator output (ms)	Comparison
Basic event sink	Periodic–periodic–event, offsets 0/6/9	40	11.000	11.000	Matches at 0.001 ms resolution
Pre-opening capture	Arrival before opening; 2 ms event window	40	11.000	11.000	Fits the upcoming window
Cross-window execution	Middle offset shifts 6 to 8 ms; unfinished execution resumes in the next window	39	25 + a; 35–36 for a in [10,11]	35.500–36.000	Within the derived completion-time range
Minimum inter-arrival	25 ms arrivals, 50 ms event service spacing	20	11 + 25k; 11–486	11–486	Recurrence agreement
Mixed chain	Periodic–event–periodic, offsets 0/6/9	40	11.000	11.000	Matches at 0.001 ms resolution

Event service and pre-opening capture

With a 25 ms cycle and offsets 0/6/9 ms, the source executes from 0 to 2 ms and the periodic middle task from 6 to 8 ms. Under the declared transfer bound of less than 1 ms, the final event arrives before 9 ms. The event task uses the service opportunity starting at 9 ms and completes at 11 ms. Thus the expected flow delay is 11 ms. In the capture case, the final window is explicitly [9,11] ms: arrival precedes opening and the 2 ms execution fits that window. This checks capture of an upcoming window, rather than arrival during an already-open window.

Boundary-sensitive execution across partition windows

Changing the middle offset from 6 to 8 ms moves middle-task completion from 8 to 10 ms. The terminal task requires 2 ms of execution. Under suspension and resumption at partition-window boundaries, unfinished execution retains its progress for the next window. Insufficient time to finish within the current window therefore does not imply that no execution occurs in that window.

Consider times relative to the corresponding source-task start. Let a denote the terminal-task start time in milliseconds. Under the declared transfer bound and immediate service upon arrival in this isolated case, a lies within [10,11] ms. The task executes for 11 − a ms before the current window closes at 11 ms, leaving 2 − (11 − a) = a − 9 ms of execution. It resumes when the next window opens at 34 ms. The completion time, and hence the flow delay measured from the source start, is therefore

t_finish = 34 + [2 − (11 − a)] = 25 + a ms.

For a in [10,11] ms, this relation gives a completion-time range of [35,36] ms. The recorded delays of 35.500–36.000 ms lie within this range. The interval [34,36] ms denotes the next partition window, whereas [35,36] ms is the completion-time range obtained after accounting for the remaining execution. Relative to the 11 ms capture case, the recorded delay increases by 24.5–25 ms. This comparison illustrates how a small phase change can defer completion to the next cycle even when part of the task executes in the current window.

Minimum inter-arrival and mixed activation

For the minimum-inter-arrival case, source instances arrive every 25 ms, while event service is spaced by 50 ms. Under the predeclared FIFO recurrence, source time is 25k, completion time is 11+50k, and delay is (11+50k)-25k = 11+25k ms. A 1000 ms horizon contains completed instances k=0,...,19, giving 20 outputs with delays from 11 to 486 ms. This case checks the activation-spacing recurrence and the completed-instance counting rule.

In the periodic–event–periodic case, source completion is 2 ms, event-middle service is 6–8 ms, and final periodic service is 9–11 ms. The resulting 11 ms delay checks dependency propagation across both activation types. Together, the five cases provide independent expectations for distinct event-related timing behaviors and their recorded instance outputs.

S14. Final-Model Ablation Settings and Detailed Results

Final-model variants share the same split, three seeds, maximum 36 epochs, early-stopping patience 12, and validation-MAE checkpoint selection. The tables give prediction metrics, parameter counts, inference times, best epochs and completed epochs.

Scheduling-edge deletion preserves scheduling descriptors in the node inputs. Direct-path deletion retains Flow inputs in graph propagation. These definitions identify the information routes changed by each ablation.

model	mae_ms	rmse_ms	r2	a10_percent	parameters	matched_single_model_latency_ms
lean	6.372	10.426	0.740	81.180	2687055	54.919
role_pooling_added	6.417	10.440	0.739	82.003	3146255	57.202
no_factorization	6.528	10.534	0.734	81.850	2214058	54.862
direct_only	9.953	13.445	0.567	63.351	137668	2.205
no_scheduling	6.452	10.719	0.725	81.557	2687055	—
no_direct	6.599	10.494	0.736	81.055	2558234	—

model	mae_ms	rmse_ms	r2	a10_percent	parameters	matched_single_model_latency_ms
no_scheduling_direct	6.625	10.682	0.727	80.999	2558234	—

variant	seed	best_epoch	epochs_completed
direct_only	20260903	28	36
direct_only	20260913	14	26
direct_only	20260923	6	18
lean	20260903	23	35
lean	20260913	20	32
lean	20260923	28	36
no_direct	20260903	31	36
no_direct	20260913	30	36
no_direct	20260923	33	36
no_factorization	20260903	24	36
no_factorization	20260913	24	36
no_factorization	20260923	25	36
no_scheduling	20260903	22	34
no_scheduling	20260913	15	27
no_scheduling	20260923	30	36
no_scheduling_direct	20260903	34	36
no_scheduling_direct	20260913	27	36
no_scheduling_direct	20260923	35	36
role_pooling_added	20260903	28	36
role_pooling_added	20260913	35	36
role_pooling_added	20260923	31	36

Interpretation of matched deletions

Scheduling-edge deletion, direct-path deletion, and their joint deletion change ensemble MAE by +0.080, +0.228 and +0.253 ms. Their paired intervals cross zero. The joint change is smaller than the sum of the two individual changes (+0.308 ms), so these observed effects are not additive. Node descriptors and retained propagation paths provide related timing information, offering a plausible explanation for the modest conditional changes; these comparisons do not separately quantify the amount of representational overlap.

The retained scheduling edges explicitly encode task–partition associations, while the direct branch gives the prediction head access to initial Flow features. Scheduling-edge deletion leaves the parameterized architecture unchanged; direct-path deletion reduces parameters by approximately 4.79%. Neither deletion improves ensemble MAE in this comparison. These results explain retaining the two paths alongside the larger simplification obtained by removing explicit role pooling.

S15. Architecture Validation and Complexity Measurements

The table reports mean best validation MAE across three seeds, selected epochs, parameter counts and matched single-model inference times for the evaluated architectures.

Role-pooling removal reduces parameters by 14.60%, from 3,146,255 to 2,687,055. Unified heterogeneous propagation uses 2,214,058 parameters, with mean best validation MAE 9.175 ms versus 8.445 ms for the retained relation-factorized model; inference medians are 54.862 and 54.919 ms. The direct-flow-only alternative has MAE 9.953 ms and removes graph propagation, graph fusion and Task auxiliary supervision.

model	best_validation_mae_mean_ms	best_epochs	parameters	matched_single_model_latency_ms
lean	8.445	23/20/28	2687055	54.919
role_pooling_added	8.375	28/35/31	3146255	57.202
no_factorization	9.175	24/24/25	2214058	54.862
direct_only	11.931	28/14/6	137668	2.205

Architecture-selection rationale

Adding explicit role pooling gives ensemble MAE 6.417 ms versus 6.372 ms for direct Flow-state readout; the paired difference is +0.046 ms with interval [-0.242,0.282] ms. Mean best validation MAE slightly favors role pooling (8.375 versus 8.445 ms), while direct readout uses fewer parameters and shorter measured inference time. The choice is therefore an accuracy–complexity trade-off, not a claim of a statistically established accuracy improvement from removing pooling.

Further removing relation factorization yields negligible measured latency saving but higher validation MAE. This favors retaining the factorized implementation. The direct-only alternative increases test MAE by 3.581 ms, with paired interval [2.404,4.740] ms, supporting the evaluated graph-plus-flow architecture. Its removal of graph fusion and auxiliary Task supervision means the contrast concerns the complete alternative architecture.
